# A prognostic nomogram for predicting recurrence-free survival of stage I–III colon cancer based on immune-infiltrating Treg-related genes

**DOI:** 10.1007/s00432-023-05187-y

**Published:** 2023-07-27

**Authors:** Longwen Xu, Mengjie Liu, Jie Lian, Enmeng Li, Chang Dongmin, Xuqi Li, Wenjuan Wang

**Affiliations:** 1 Department of Medical Oncology, The First Affiliated Hospital of Xi’an Jiaotong University, Xi’an, Shaanxi China; 2 Department of General Surgery, The First Affiliated Hospital of Xian Jiaotong University, Xi’an, Shaanxi China; 3 Department of Pathology, The First Affiliated Hospital of Xian Jiaotong University, Xi’an, Shaanxi China; 4 Department of Surgical Oncology, The First Affiliated Hospital of Xi’an Jiaotong University, Xi’an, Shaanxi China

**Keywords:** Colon cancer, Tregs (regulatory T cells), Nomogram, Recurrence, Tumor-immune microenvironment

## Abstract

**Purpose:**

A high postoperative recurrence rate seriously impedes colon cancer (CC) patients from achieving long-term survival. Here, we aimed to develop a Treg-related classifier that can help predict recurrence-free survival (RFS) and therapy benefits of stage I–III colon cancer.

**Methods:**

A Treg-related prognostic classifier was built through a variety of bioinformatic methods, whose performance was assessed by KM survival curves, time-dependent receiver operating characteristic (tROC), and Harrell’s concordance index (C-index). A prognostic nomogram was generated using this classifier and other traditional clinical parameters. Moreover, the predictive values of this classifier for immunotherapy and chemotherapy therapeutic efficacy were tested using multiple immunotherapy sets and R package “pRRophetic".

**Results:**

A nine Treg-related classifier categorized CC patients into high- and low-risk groups with distinct RFS in the multiple datasets (all *p* < 0.05). The AUC values of 5-year RFS were 0.712, 0.588, 0.669, and 0.662 in the training, 1st, 2nd, and entire validation sets, respectively. Furthermore, this classifier was identified as an independent predictor of RFS. Finally, a nomogram combining this classifier and three clinical variables was generated, the analysis of tROC, C-index, calibration curves, and the comparative analysis with other signatures confirmed its predictive performance. Moreover, KM analysis exhibited an obvious discrepancy in the subgroups, especially in different TNM stages and with adjuvant chemotherapy. We detected the difference between the two risk subsets of immune cell sub-population and the response to immunotherapy and chemotherapy.

**Conclusions:**

We built a robust Treg-related classifier and generated a prognostic nomogram that predicts recurrence-free survival in stage I–III colon cancer that can identify high-risk patients for more personalized and effective therapy.

**Supplementary Information:**

The online version contains supplementary material available at 10.1007/s00432-023-05187-y.

## Introduction

Colon cancer accounted for the third most new cancer cases globally in 2020 and was the second leading cause of cancer-related deaths (Sung et al. 2021). Surgery and chemotherapy are currently the primary treatment methods for stage I–III colon cancer. The American Joint Commission on Cancer (AJCC) tumor-node-metastasis (TNM) staging system is also the primary basis for evaluating the prognosis after the radical operation and determining the follow-up treatment plan (Wang et al. 2019a, b). However, due to the high heterogeneity of tumors, the prognosis of patients with the same stage or clinical characteristics differed distinctively. Therefore, a better prognostic indicator or predictive model is needed to identify colon cancer behavior.

Colon cancer is the outcome of genomic instability caused by the accumulation of numerous oncogene mutations or the inactivation of tumor-suppressor genes (Lin et al. 2021). With our ongoing knowledge of tumors, cognition is continuously evolving. The tumor microenvironment, the environment in which cancer develops, has also received significant attention. Immunologically, TME is mainly composed of tumor promoting components such as cancer-associated fibroblasts (CAFs), tumor blood vessels, M2 tumor-associated macrophages (TAMs), T helper-2 (Th2) cell factors, and tumor inhibiting components such as T cells, natural killer (NK), M1 TAMs, Th1, cytokines and other components (Thakkar et al. 2020). When the tumor promoting components are overwhelming in quantity and function, TME shows a profound immunosuppressive state (Ma et al. 2020). Previous studies indicated that the immune response within the tumor microenvironment has an important effect on the occurrence and development of colon cancer and the response to immunotherapy, including exploration of Treg (regulatory T cells) cell function (Orhan et al. 2020). Treg is a kind of T cell subset essential to control self-tolerance and inflammatory response, divided into CD4 + Treg and CD8 + Treg (Tanaka et al. 2017). Immunosuppression and immune incompetence are the two functional characteristics of Treg cells (Wing et al. 2019). The role of Treg in tumorigenesis and development involves the regulation of tumor immunity (Dees et al. 2021), angiogenesis (Kajal et al. 2021) and tumor cell proliferation (Thakkar et al. 2020), and interacts with various components in tumor microenvironment. Several recent studies demonstrated that Treg components in patients’ peripheral blood or local tumors increased significantly in liver cancer (Li et al. 2014), ovarian cancer (Winkler et al. 2015), breast cancer (Wang et al. 2019a, b), acute and chronic lymphocytic leukemia (Niedzwiecki et al. 2019), and nasopharyngeal carcinoma (Liu et al. 2021). In the study of colorectal cancer, it was found that the peripheral blood Tregs of patients with advanced cancer were significantly higher than those of patients with early stage (Krijgsman et al. 2019). Previous studies showed that Treg is associated with poor prognosis in cervical cancer (Punt et al. 2015), lung cancer (Shimizu et al. 2010), breast cancer (Wang et al. 2019a, b), melanoma and other tumors (Shang et al. 2015); however, its role in colon cancer and its impact on the prognosis remain unclear.

In this study, we integrated five cohorts from TCGA (The Cancer Genome Atlas) and GEO (Gene Expression Omnibus) to explore the potential role of Treg cells in colon cancer using bioinformatics models. Weighted gene co-expression network analysis (WGCNA) was performed to identify the most significant module and candidate genes related to Tregs. Furthermore, we developed a novel Treg-related classifier and constructed a robust nomogram to predict recurrence-free survival in colon cancer patients with stage I–III. Moreover, we found differences in immune cell sub-population and immunotherapy and chemotherapy response between the two risk subsets, which may explain for the disparity in RFS between the two subsets.

## Materials and methods

### Dataset source and processing

A total of 1194 stage I–III CC patients with clinical data of TNM stage I–III, RFS (recurrence-free survival) and RFS status were retrieved from different platforms. We downloaded the microarray dataset GSE39582 from GEO (http://www.ncbi.nlm.nih.gov/geo/) as the training set. This dataset was produced by a Affymetrix Human Genome U133 Plus 2.0 Array and included 485 stage I–III CC patients meeting the standard. The first validation set included datasets GSE37892, GSE33113, and GSE17536 from the same chip platform (GPL570, Affymetrix HG-U133 Plus 2.0 Array) and contained a total of 392 stage I–III CC patients who fulfilled inclusion criteria. The batch effects of the first validation set (1st validation set) were removed using ComBat method by R package “sva” (Leek et al. 2012). RNA-sequencing data of 317 stage I–III CC patients as the second validation set (2nd validation set) were obtained from The Cancer Genome Atlas (TCGA).

### Estimation of immune-infiltrating cells

We used CIBERSORTx (https://cibersortx.stanford.edu/) to estimate the levels of 22 tumor-infiltrating immune cells using the mRNA expression data. This online tool uses a deconvolution algorithm to impute gene expression profiles and estimates the type and abundance of immune cells.

### Construction of the co-expression network

We used the R package “WGCNA (Weighted Correlation Network Analysis)” (Langfelder et al. 2008) to construct a weight co-expression network with the 16,393 gene expression values in the training cohort. The purpose of this analysis method was to find the gene modules that were co- expressed, and to explore the relationship between the modules and Tregs, as well as the target genes in the modules.

The levels of 22 immune-infiltrating cells were used as sample traits. When setting the index of scale-free topologies as 0.90, a scaleless network was successfully developed with an optimal soft-threshold power (*β* = 8). We then divided genes with similar expression patterns into the same module (minimum size = 100) using the “dynamic tree cutting” algorithm. Furthermore, to select the remarkable modules, Pearson’s test was used to evaluate the relationship between the module eigengenes and the level of the 22 immune cell types. Finally, the “regulatory T cells (Tregs)” subtype was selected and further study of the Treg-related module was conducted.

### Construction and verification of the prognostic Treg-related gene prediction model

Univariate Cox regression and KM survival analyses were performed to estimate the hazard proportions for genes with the highest correlation with Tregs (grey module). To further screen the prognosis of Treg-related genes with the best predictive performance, the “glmnet” R package (Friedman et al. 2010) was used to perform the LASSO regression analysis with tenfold cross-validation. Based on the AIC (Akaike information criterion) value on the prognosis of Treg-related genes, the bi-directional stepwise multivariate Cox regression was used for choosing the ones that minimize the AIC to obtain the best model fit. A prognostic Treg-related risk score model for CC patients was then established based on the combination of the multiplication of the multivariate Cox regression coefficient by its corresponding normalized mRNA expression value. The risk score = ∑ (the multivariate Cox coefficient of Treg-related genes × matching normalized expression level of these genes). We computed the risk scores of each CC patient. Then, we divided them into high- and low-risk subsets according to the cutoff value determined via ROC curve analysis using the R package “survminer”. The KM curve was then performed to estimate the disparity in RFS between low- and high-risk subsets using the log-rank test. The prognostic ability of the Treg-related classifier was explored with analysis of the C-index and the ROC curve. We also used the similar methods to verify the prognostic performance of the classifier constructed by the training cohort in the 1st, 2nd, and entire validation cohorts.

Furthermore, based on univariate Cox regression and multivariate Cox regression analyses, we further confirmed whether the predictive performance of the Treg-related classifier could be an independent prognostic factor compared to other clinic factors for CC patients in multiple cohorts. Finally, following the multivariate Cox regression analysis, risk score and traditional clinical factors were used to generate the nomogram using “rms,” “foreign,” and “survival” R packages. C-index, tROC curve, and calibration plots of the nomogram for 1-, 3-, and 5-year RFS plotted were applied to elucidate the accuracy of actual observed rates with the predicted survival probability. The “timeROC” R package was utilized to perform the tROC analyses.

### Construction and validation of nomogram model

Based on the risk score with traditional clinical parameters including age, gender, and stage, a prognostic CC nomogram model was constructed by the “rms” R package to apply the clinical application of Treg-related genes. The ROC analysis, C-index, and calibration were used to evaluate and compare the accuracy of the nomogram.

### Functional enrichment analysis

To probe underlying functions of differentially expressed Treg-related genes and risk model and screen the critical altered signaling pathways, the R package “clusterProfiler” (Yu et al. 2012) was utilized to perform Gene Ontology (GO), Kyoto Encyclopedia of Genes and Genomes (KEGG) pathways analysis and Gene Set Enrichment Analysis (GESA) between the two subsets in the 2nd validation cohort from the TCGA-COAD project. The “c2.cp.kegg.v7.0.symbols.gmt” KEGG gene set was acquired from MSigDB (The Molecular Signatures Database). The thresholds were the nominal p value (NOM-P) for gene sets < 0.05, the absolute normalized enrichment score |NES|> 1.7 and the false discovery rate (FDR) < 0.1.

### Prediction of immunotherapy and chemotherapy efficacy

The cytotoxic activity (CYT) (Rooney et al. 2015), T cell-inflamed gene expression profile (GEP) (Cristescu et al. 2018), and the Tumor Immune Dysfunction and Exclusion (TIDE) algorithm (Jiang et al. 2018) were applied to predict immunotherapy efficacy between the different risk groups in the entire validation dataset. Moreover, we abstracted RNA-seq expression and clinical data from three online available sets of patients receiving anti-PD-1/PD-L1 antibody therapy, including IMvigor210 set (advanced urothelial cancer, *n* = 348), GSE135222 set (advanced non-small cell lung carcinoma, *n*  = 27), and GSE162137 set (cutaneous T cell lymphoma, *n* = 64), to evaluate the prognostic value of Treg-related classifier in predicting immunotherapy response. Data of IMvigor210 set was obtained from “http://research-pub.gene.com/IMvigor210CoreBiologies” via the “IMvigor210CoreBiologies” R package and the data of GSE135222 set and GSE162137 set were download from the GEO database. We then divided patients into two risk groups according to our Treg-related classifier and the survival difference and response rate between these two risk groups were evaluated. More than that, the drug sensitivity to chemo-agents was predicted using the R package “pRRophetic version 0.5” to count the half-maximal inhibitory concentration (IC50) of six common chemotherapy (cisplatin, gemcitabine, paclitaxel, docetaxel, doxorubicin, and rapamycin) in the training cohort (Geeleher et al. 2014). The difference in IC50 of these drugs between risk groups was conducted using the Wilcoxon rank-sum test.

### Statistical analysis

Software R (version 4.1.0) was used to performed all data analyses. Wilcoxon and Chi-square tests assessed the relationship between the risk score and clinical parameters. The Kaplan–Meier (KM) survival analysis was performed using the log-rank test. Two-tailed *p* < 0.05 was considered statistically significance. The detailed versions of R packages included in this article were followed: sva (version 3.42.0), WGCNA (version 1.72–1), glmnet (version 4.1–7), rms (version 6.7–0), foreign (version 0.8–84), survival (version 3.5–5), timeROC (version 0.4), clusterProfiler (version 4.9.0.002), IMvigor210CoreBiologies (version 1.0.0) and pRRophetic (version 0.5).

## Results

### Identification of Hub Treg-related module by WGCNA

**Fig. 1:**
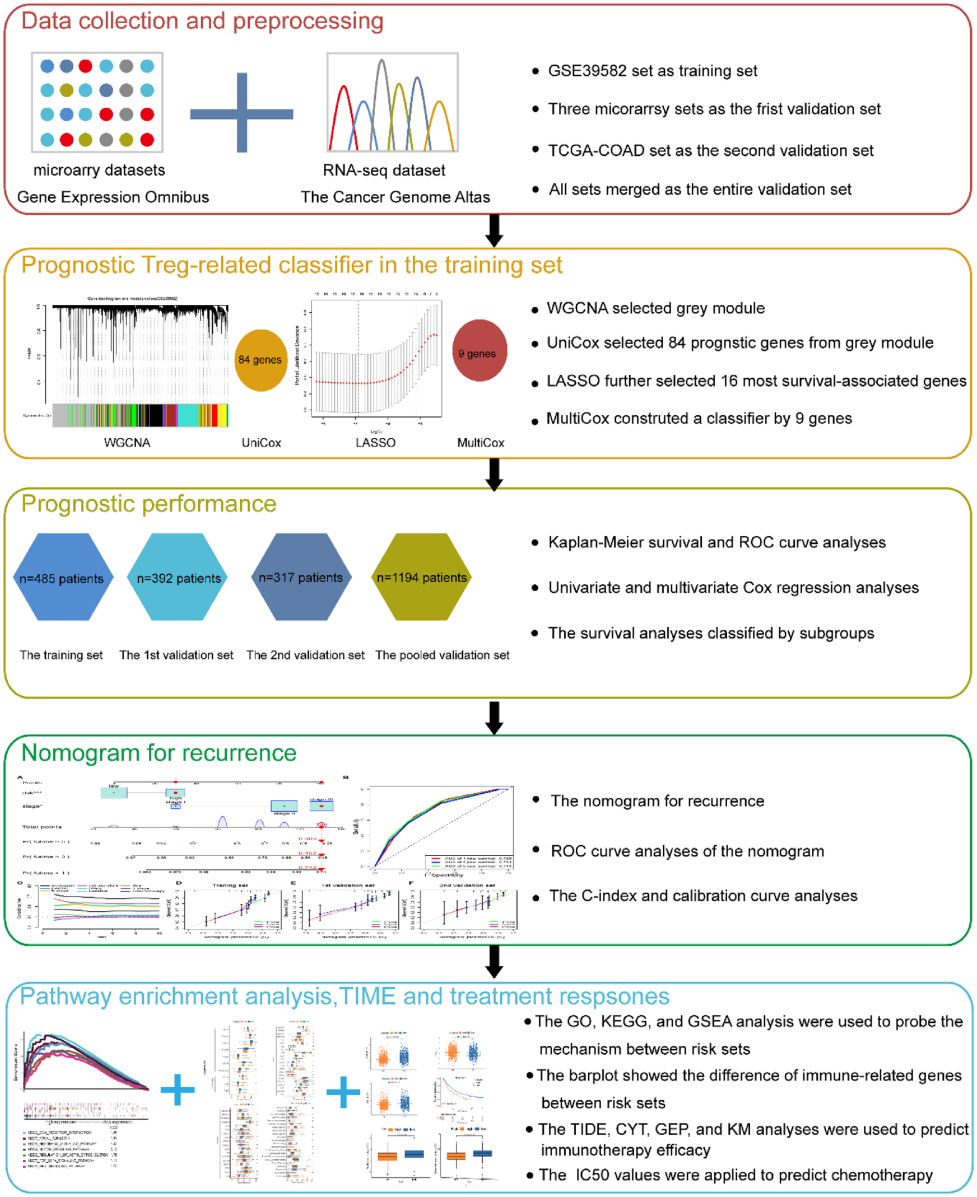
HYPERLINK "sps:id::fig1||locator::gr1||MediaObject::0" The study design and workflow

The study design and workflow are depicted in Fig. 1. As mentioned above, we collected 1194 patients diagnosed with colon cancer with TNM stage range from I to III from the GEO and TCGA databases. A total of 485 colon cancer patients from the GES39582 set were set as the training cohort, 392 CC patients from 3 microarrays sets (GSE37892, GSE33113, and GSE17536) from GEO were integrated into the first (1st) validation set, 317 CC patients from TCGA-COAD project were grouped into the second (2nd) validation set, and the total CC patients (*n* = 1194) were merged as the entire validation set. A combat method was applied to remove batch effects from these merged sets (Supplementary Fig. 1).

We then extracted the training cohort's mRNA gene expression profiles for 485 CC samples. The abundance of 22 tumor-infiltrating immune cell subtypes for these CC tissues was next counted using the CIBERSORT algorithm. The expression profiles of the 16,393 genes were then used to build the gene co-expression network of CC via WGCNA method. The training cohort grouped samples based on the Pearson’s correlation coefficients and average linkage values. Our scale-free network was constructed using *β* = 8 with scale-free R2 = 0.9 (Supplementary Fig. 2A–B). Then, ten gene modules were conducted by hierarchical clustering tree (Supplementary Fig. 2C). The result showed that the grey gene modules including 4813 genes were strongly related to T cells regulatory (Tregs, R2 = 0.34, *P* = 3e−15) (Supplementary Fig. 3).

### Establishment of the prognostic Treg-related classifier in the training dataset

**Fig. 2:**
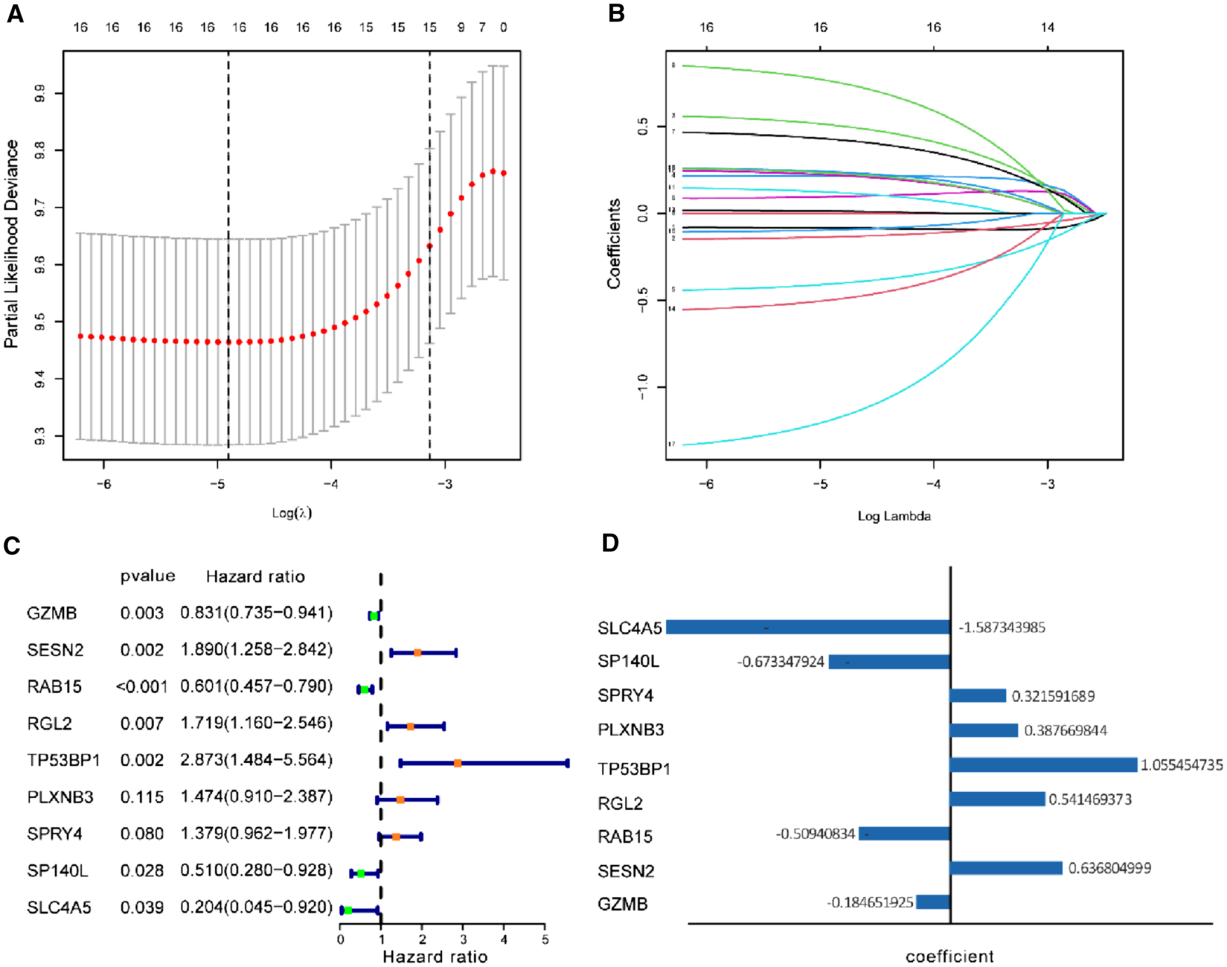
Construction of Treg-related genes signature. **A** Tenfold cross-validation with minimum criteria for tuning parameter selection (*λ*) in the LASSO model. **B** LASSO coefficient profiles of the Treg-related genes. The dotted line indicates the value chosen by threefold cross-validation. **C** Multivariable Cox regression analysis of these Treg-related genes adopted in the signature. **D** The coefficient of these Treg-related genes using multivariable Cox regression analysis

**Fig. 3:**
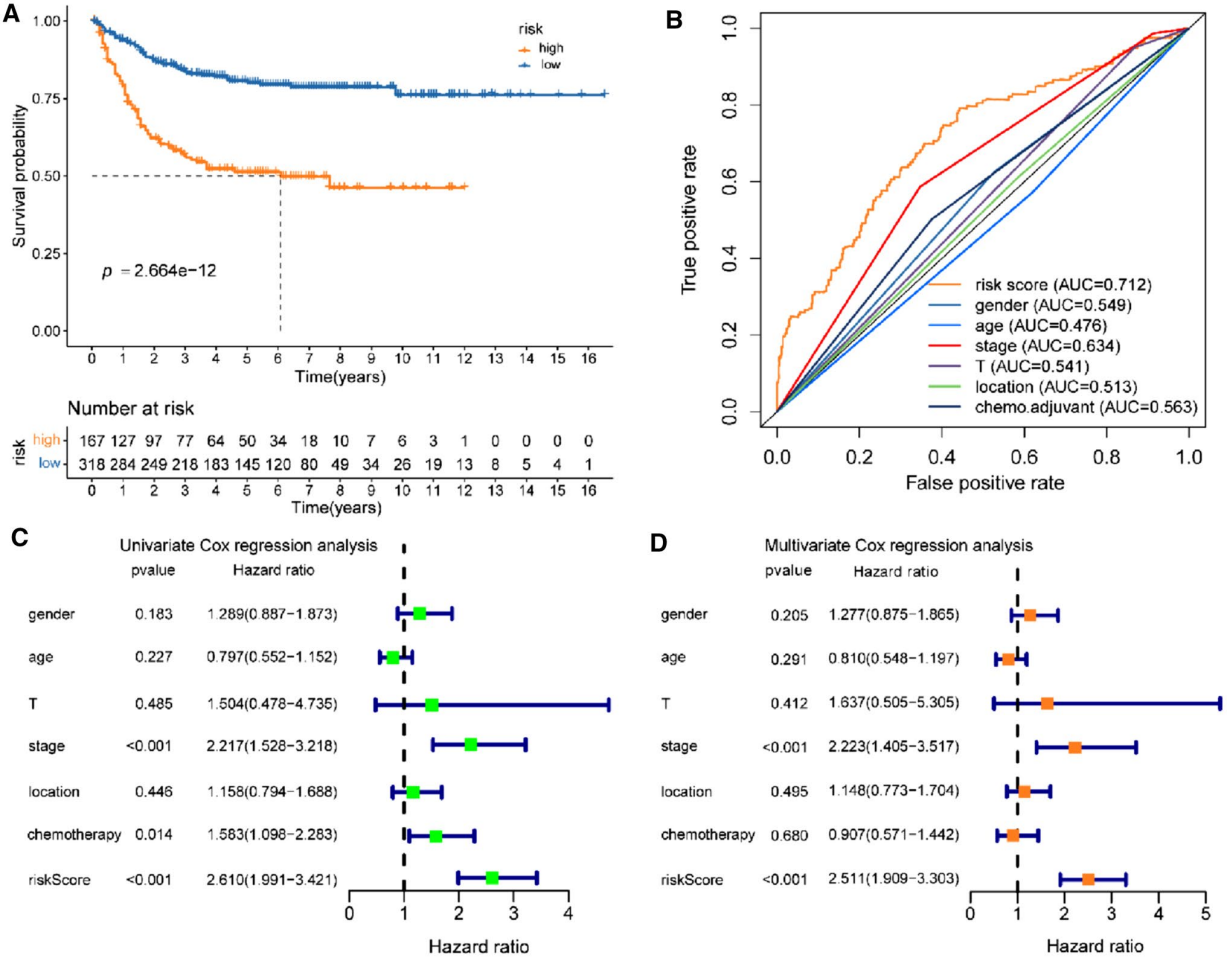
Kaplan–Meier (KM), time-dependent ROC (tROC) and cox regression analysis of the Treg-related risk score model in the training set. **A** KM curve of the Treg-related signature for recurrence-free survival (RFS). **B** ROC analysis of the Treg-related signature for 5-year RFS. **C** Univariate Cox regression analysis of the Treg-related signature. **D** Multivariate Cox regression analysis of the Treg-related signature

In the training set of 485 CC stage I–III patients, 84 Treg-related genes were significantly linked with recurrence-free survival (RFS) after performing univariate Cox regression and Kaplan–Meier survival analysis in 4813 genes of the grey module. These RFS-associated Treg-related genes were filtered into an analysis of Lasso penalized Cox regression (Fig. 2A, B) and multivariate Cox regression (Fig. 2C). We derived a Treg-related classifier based on the nine most likely RFS-associated Treg-related genes to count the risk score of each CC patient. The formula of risk score based on the regression coefficients of the nine mRNAs weighted by their expression levels was followed: risk score = (0.637 × level of SESN2 expression + 0.541 × level of RGL2 expression + 1.055 × level of TP53BP1 expression + 0.388 × level of PLXNB3 expression + 0.321 × level of SPRY4 expression − 0.185 × level of GZMB expression − 0.509 × level of RAB15 expression − 0.673 × level of SP140L expression − 1.587 × level of SLC4A5 expression) (Fig. 2D). The optimal cutoff score (3.600) was computed by the “survminer” package in the training set. The cutoff score then classified CC patients in the training dataset into high-risk-and low-risk subsets. The rates of recurrence-free survival (RFS) for patients within the high-risk subset were 56.0% at 3 years, 51.2% at 5 years, and 46.2% at 7 years, compared with 83.3%, 80.1%, and 76.0% in patients within the low-risk subset, respectively (log-rank *P* = 2.664e−12, Fig. 3A). After adjusting the clinicopathological features by analysis of univariate and multivariate regression, the risk score based on the nine Treg-related genes found to be an independent factor for predicting RFS (all HR > 1 and *p* value < 0.05) in the training dataset (Fig. 3C, D). Furthermore, a 5-year RFS ROC curve analysis was performed to measure the predictive performance of the Treg-related classifier. We found our signature exhibited the highest AUC value of 0.712, which was better than that of gender (AUC = 0.549), age (AUC = 0.476), T stage (AUC = 0.541), TNM stage (AUC = 0.634), location (AUC = 0.513), and chemotherapy (AUC = 0.563), suggesting that the powerful prediction of recurrence than the other clinical variables (Fig. 3B).

### Verification of the Treg-related signature in the validation datasets and the entire dataset

To confirm the predictive ability of our Treg-related classifier, we utilized the 1st validation set (*n* = 392 patients), the 2nd validation set (*n* = 317 patients), and the entire set (*n* = 1194 patients), including the training set and other two validation sets, to test the reliability of the predictive capacity of the signature.

**Fig. 4:**
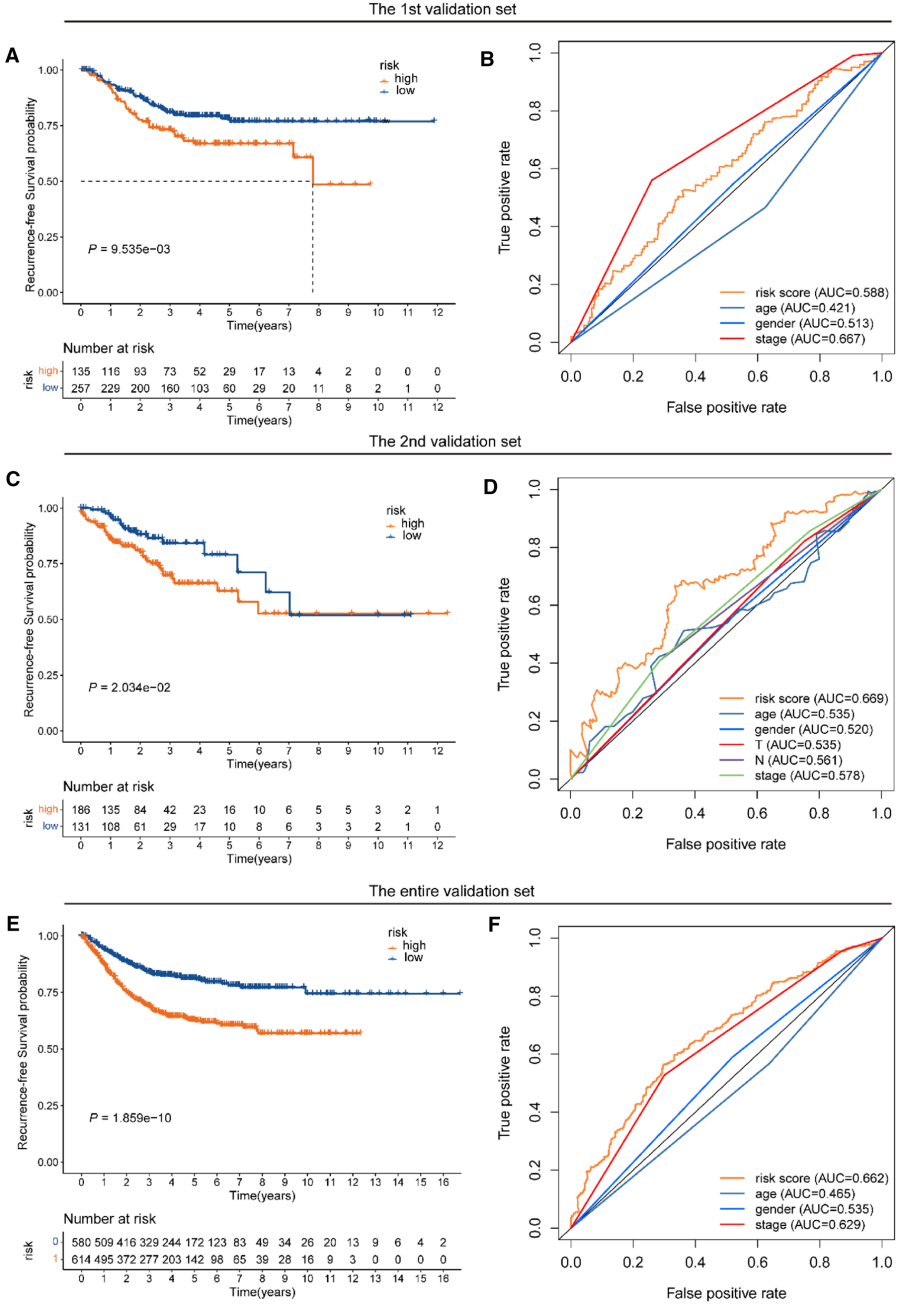
KM and ROC analysis of the Treg-related signature in validation sets. **A, B** KM and ROC curve of the signature in the 1st validation set. **C, D** KM and ROC curve of the signature in the 2nd validation set. **E, F** KM and ROC curve of the signature in the entire validation set

**Fig. 5:**
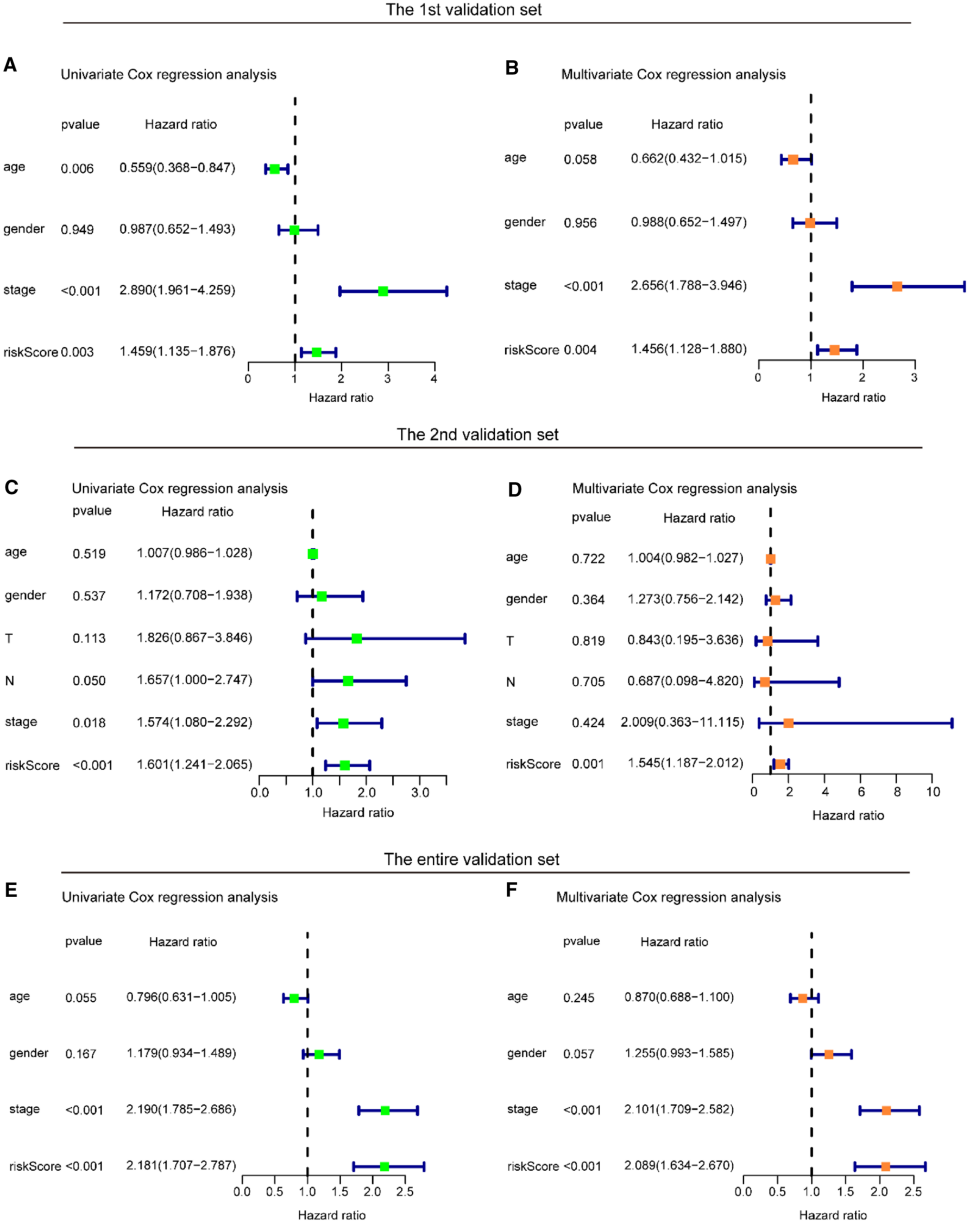
Univariate and multivariate Cox regression analyses of the Treg-related signature in validation sets. **A, B** Univariate and multivariate Cox regression analyses of the signature in the 1st validation set. **C, D** Univariate and multivariate Cox regression analyses of the signature in the 2nd validation set. **E, F** Univariate and multivariate Cox regression analyses of the signature in the entire validation set

Using the above Treg-related classifier, the risk scores of CC patients from the validation sets were counted. Then, in the 1st validation set, 135 patients were divided into a high-risk subset and 257 patients into a low-risk subset, and the patients from the 2nd validation set were grouped as a high-risk subset (*n* = 186) and a low-risk subset (*n* = 131) based on the above cutoff point. In the 1st validation set, the rates of RFS for patients from high-risk group were 72.4% at 3 years, 61.0% at 5 years, and 61.0% at 7 years, compared with 80.2%, 76.7%, and 76.7% in patients from the low-risk group, respectively (log-rank *p* = 9.535e−03, Fig. 4A). In the 2nd validation set, the rates of RFS for patients from the high-risk group were 67.7% at 3 years, and 56.9% at 5 years, compared with 79.6%, and 72.3% in patients from the low-risk group, respectively (log-rank *p* = 2.034e−02, Fig. 4C). After adjusting the clinicopathological features by analysis of univariate and multivariate regression, the risk score based on the nine Treg-related genes was found to be an independent factor for predicting RFS (all HR > 1 and *p* value < 0.05) in all validation datasets (Fig. 5A–D). Moreover, we found that AUC points for the 5-year RFS of our signature were 0.588 and 0.669, respectively, which ranked as the second and first predictive accuracy among the other clinical variables in the 1st and 2nd validation sets (Fig. 4B, D).

Following the same analyses as above, the Treg-related risk signature yielded similar results. A total of 1194 patients from the entire set were separated into the low-risk group (*n* = 580) and high-risk group (*n* = 614) with significantly distinct RFS (Fig. 4E). The classifier constructed with the nine Treg-related genes also proved to be an independent factor for predicting RFS (HR > 1 and *p* value < 0.05, Fig. 5E, F). The Treg-related risk model showed the highest prediction accuracy (AUC value = 0.662) among the other clinical variables (Fig. 4F), indicating that our Treg-related signature has a powerful and robust predictive accuracy for predicting RFS.

### Comprehensive insights into the Treg-related classifier involved in colon cancer

**Fig. 6:**
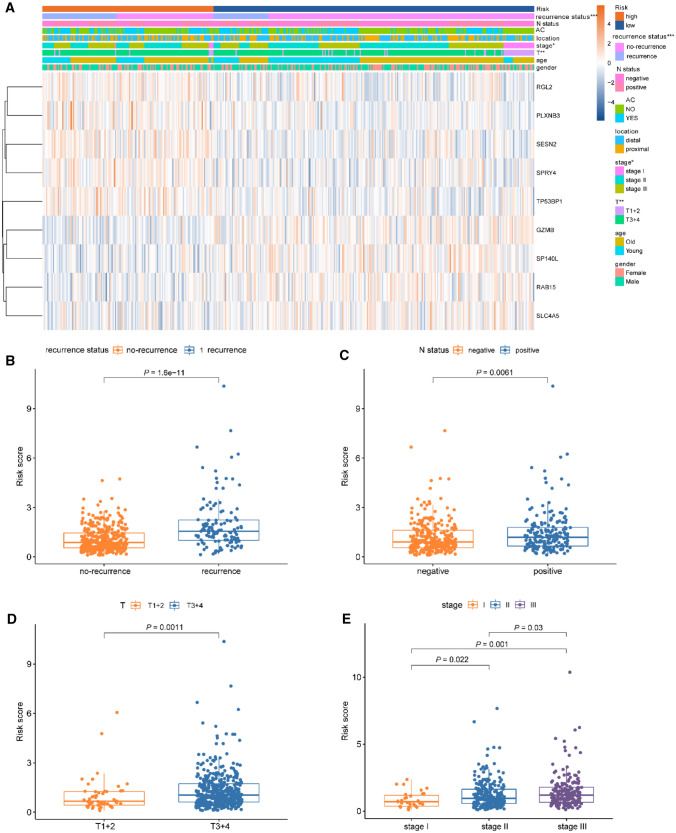
The correlation between the Treg-related signature and clinical variables. **A** The heatmap revealed the association of the Treg-related signature and the clinical variables (chemotherapy, IDH status, radiotherapy, grade, gender, age and survival status) in the training set. **B–E** The box plots displayed the relationship between risk score and clinical features

To elucidate the clinical impact of the Treg-related classifier in CC patients, we analyzed the association of the classifier with clinical variables in the training set. The Treg-related signature was significantly associated with recurrence-free survival status, N status, T stage, and TNM stage, except for age, gender, and adjuvant chemotherapy (Fig. 6A). We further analyzed the risk scores in different subsets grouped by recurrence-free survival status, N status, T stage, and TNM stage. Compared to the non-recurrence group, patients with recurrence had elevated risk scores (Fig. 6B). Regarding N status, the risk scores in the negative group were lower than those in the positive group (Fig. 6C). Patients with T3 + 4 exhibited a higher score than those with T1 + 2 (Fig. 6D). Based on the stage of TNM, the risk scores increased in the stages II and stage III compared to stage I (Fig. 6E). These findings indicated that the risk score was positively related to aggressive clinicopathological subtypes (such as positive lymph node metastases and higher T stage). We then investigated the prognostic effects of our Treg-related signature in different subsets grouped by clinicopathological variables. Patients with high-risk scores had decreased RFS in both age subsets (Supplementary Fig. 4A, B, *p* < 0.001). Similar significant findings were revealed in the gender groups (Supplementary Fig. 4C, D, *p* < 0.001), distal and proximal groups (Supplementary Fig. 4E, F, *p* < 0.001), negative node metastasis and positive node metastasis groups (Supplementary Fig. 4G, H, *p* < 0.0001), T stage subtypes (Supplementary Fig. 4I, J, *p* < 0.0001), and different TNM stages (Supplementary Fig. 4K, M, *p* < 0.05).

**Fig. 7:**
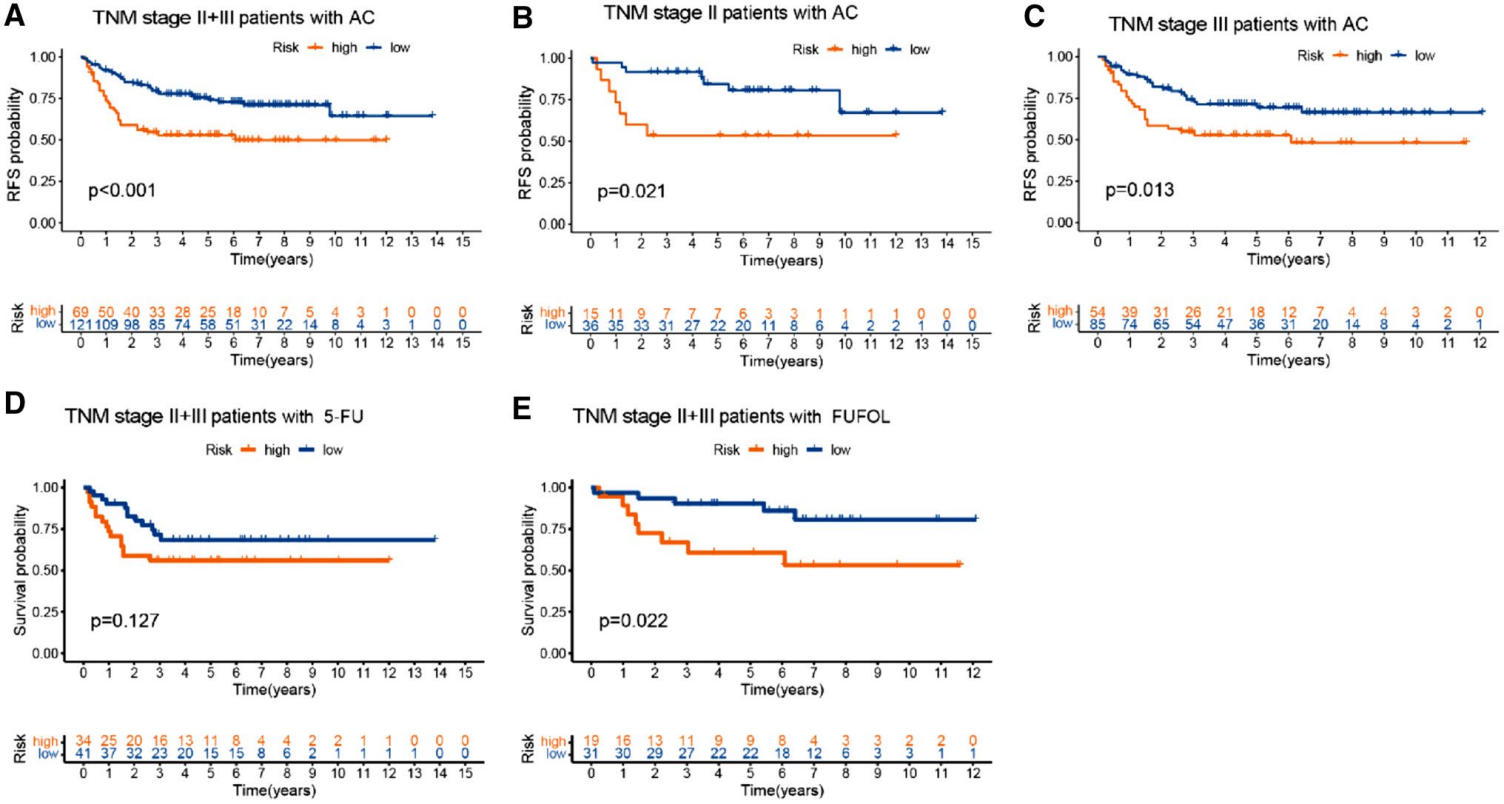
Adjuvant chemotherapy (AC) benefits stratified by different TNM stage. KM survival curves for CC patients receiving with AC subset, which were stratified by different TNM stage. **A** TNM stage II + III; **B** TNM stage II; **C** TNM stage III; D TNM stage II + III with 5-FU; E TNM stage II + III with FUFOL. *5-FU* 5-Fluorouracil, *FUFOL* Fluorouracil and Leucovorin

Due to the inadequate response to adjuvant therapy following radical surgery, the survival duration of certain patients tends to be shorter. Thus, we investigated whether our Treg-related signature possesses the potential to predict the response to clinical intervention in colon cancer. The findings derived from the training cohort revealed an unexpected observation, suggesting a remarkable link between high-risk scores and therapy resistance to adjuvant chemotherapy, even among distinct TNM stages (Fig. 7A–C, p < 0.05). Furthermore, when focusing on particular therapeutic approaches, our analysis revealed that patients within the high-risk group exhibited significantly lower responsiveness 5-Fluorouracil (5-FU, Fig. 7D, p = 0.127) and Fluorouracil and Leucovorin (FUFOL, Fig. 7E, p = 0.022).

### Constructing a prognostic nomogram for recurrence

**Fig. 8:**
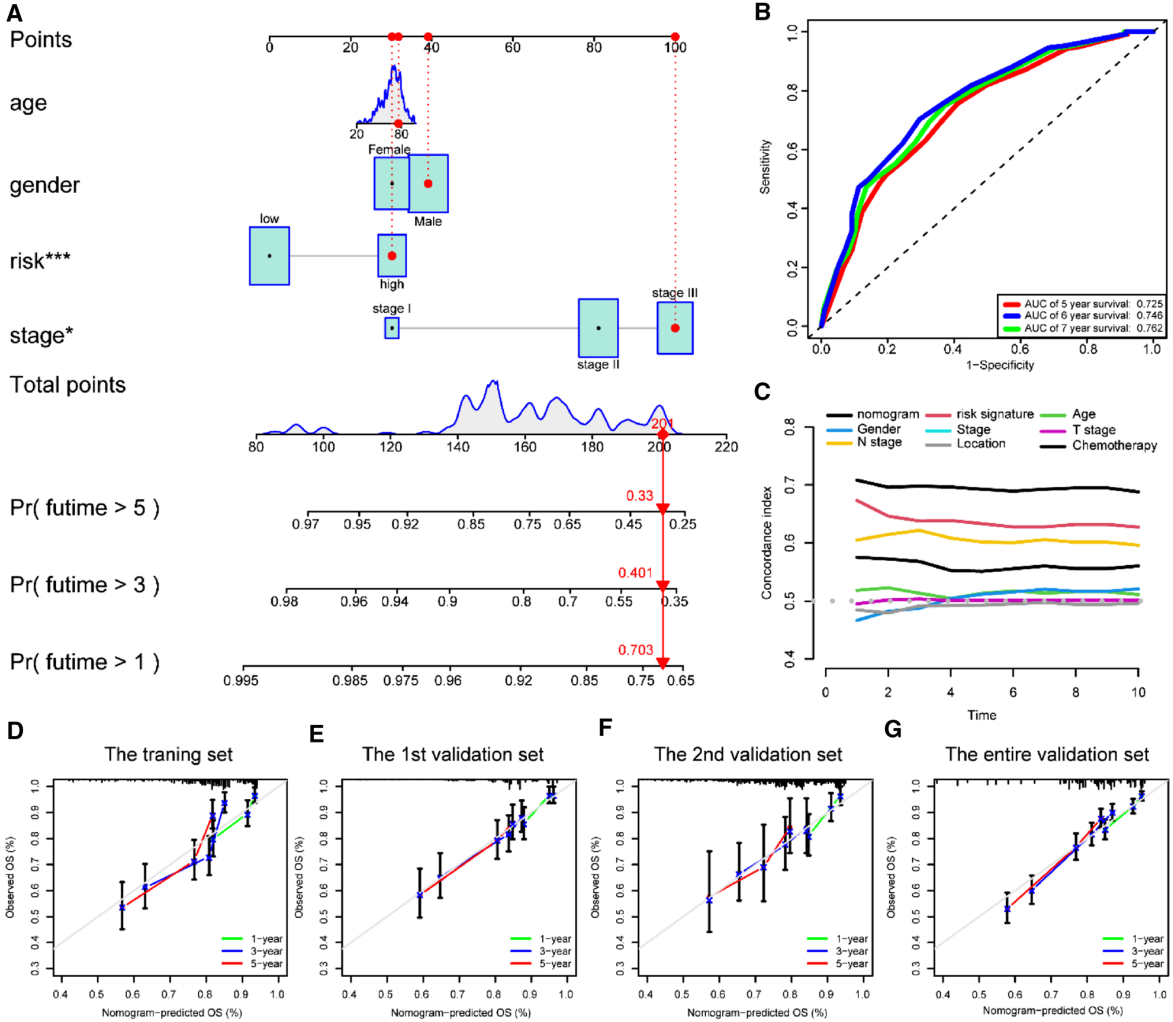
A nomogram was constructed to predict the RFS. **A** A nomogram for predicting 1-, 3- and 5-year RFS with risk levels and three clinical variables. **B** 5-, 6- and 7-year ROC curves of the nomogram for RFS predictions. **C** The C-index of the nomogram, risk signature and other clinical variables. **D** Calibration plots of nomogram for predicting probabilities of 1-year, 3-year, and 5-year RFS of CC patients in the training dataset. **E** Calibration plots of nomogram for predicting probabilities of 1-year, 3-year, and 5-year RFS of CC patients in the 1st validation dataset. **F** Calibration plots of nomogram for predicting probabilities of 1-year, 3-year, and 5-year RFS of CC patients in the 2nd validation dataset. **G** Calibration plots of nomogram for predicting probabilities of 1-year, 3-year, and 5-year RFS of CC patients in the entire validation dataset. The blue line indicates actual survival

We constructed a prognostic nomogram to predict the 1-, 3-, and 5-year RFS probability of CC patients in the training dataset by combining the Treg-related classifier with three clinicopathological variables shared in the training dataset and the other validation datasets (Fig. 8A). The AUC points of the nomogram for 5-, 6-, and 7-year RFS predictions were 0.725, 0.746, and 0.762, respectively (Fig. 8B). The C-index indicated that the nomogram had the highest predictive accuracy of RFS than other clinicopathological parameters (Fig. 8C). Moreover, the calibration curves also confirmed a good consistency between predicted and observed scores in terms of probabilities of 1-, 3-, and 5-year RFS (Fig. 8D). Similar results of calibration curves of nomogram were also found in the 1st, 2nd, and entire validation datasets (Fig. 8E-G). Together, our nomogram was clinically suitable for clinical practice based on these findings.

### A comparative analysis of prognostic signatures for RFS in stage I–III colon cancer

**Fig. 9:**
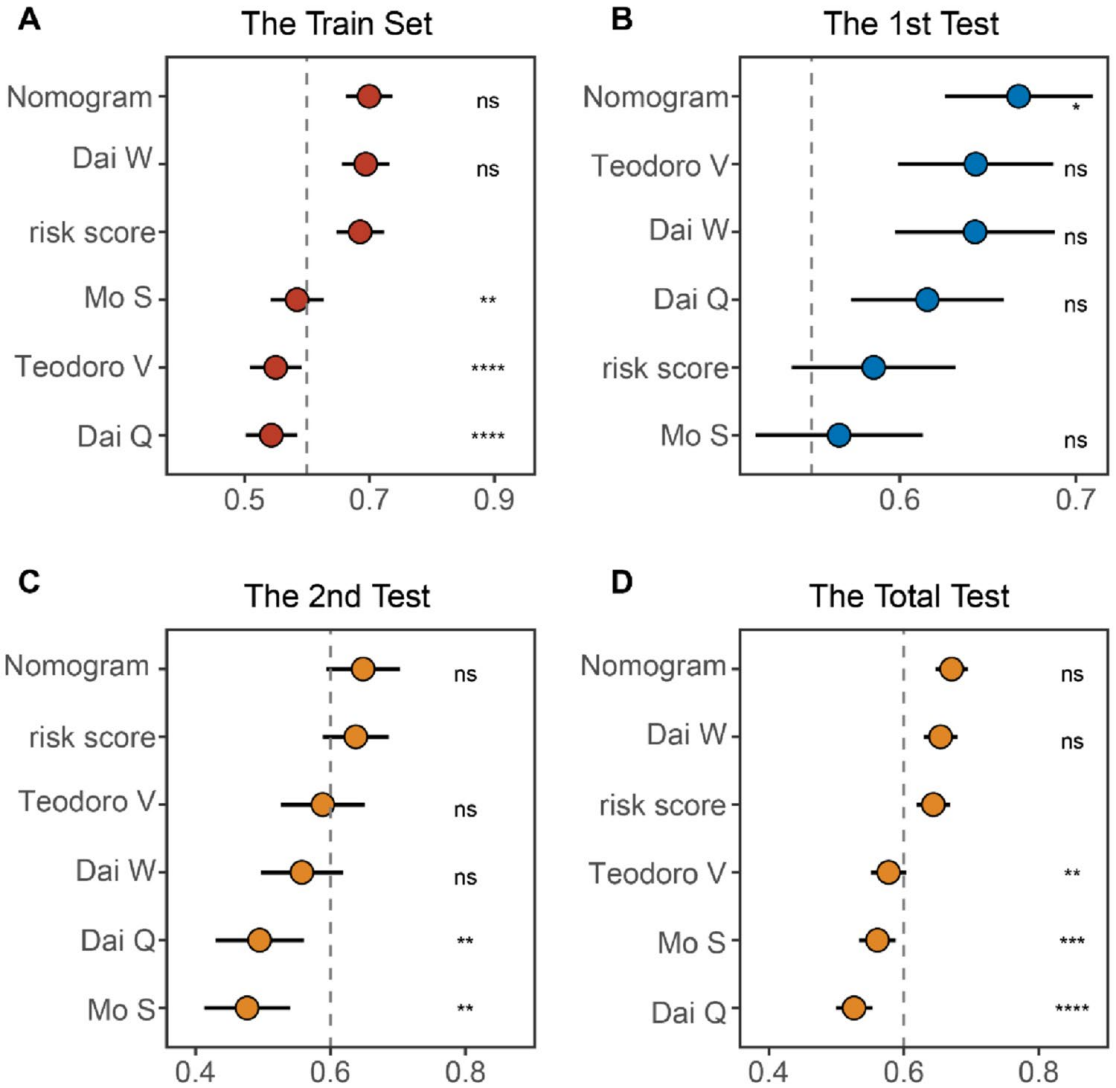
Comparison of prognostic signatures for RFS in stage I–III colon cancer. C-index analysis risk score and nomogram and 4 published signatures in the training set (*n* = 485), 1st test set (*n* = 392), 2nd test set (*n* = 317), and total set (*n* = 1194). Statistic tests: two-sided z-score test. *Ns* not significant; *P < 0.05; **P < 0.01; ***P < 0.001; ****P < 0.0001

To compare the performance of nomogram and Treg-related classifier with other signatures, we comprehensively extracted genes from 11 relevant models, with or without corresponding coefficients. However, after intersecting 16,352 common genes in the datasets of this article with genes included in these signatures, only 4 signature’s genes were all expressed in these datasets, and retained for further analysis (Supplement Table 1). We first assessed whether these signatures have statistical significance in all datasets using unicox analysis. The findings of univariate Cox regression showed that only our risk score counted by Treg-related classifier, nomogram constructed by integrated risk, age, gender, and TNM stage, and 4-gene signature of Teodoro V had consistent statistical significance in the training, 1st validation, 2nd validation, and entire validation sets (Supplement Table 2). Afterwards, we use the C-index to compare the predictive power for predicting RFS between our signatures and other signatures. Notably, our nomogram exhibited superior accuracy than the other models in all sets, revealing the robustness of the nomogram (Fig. 9A–D).

### Pathway enrichment and functional annotation analysis

**Fig. 10:**
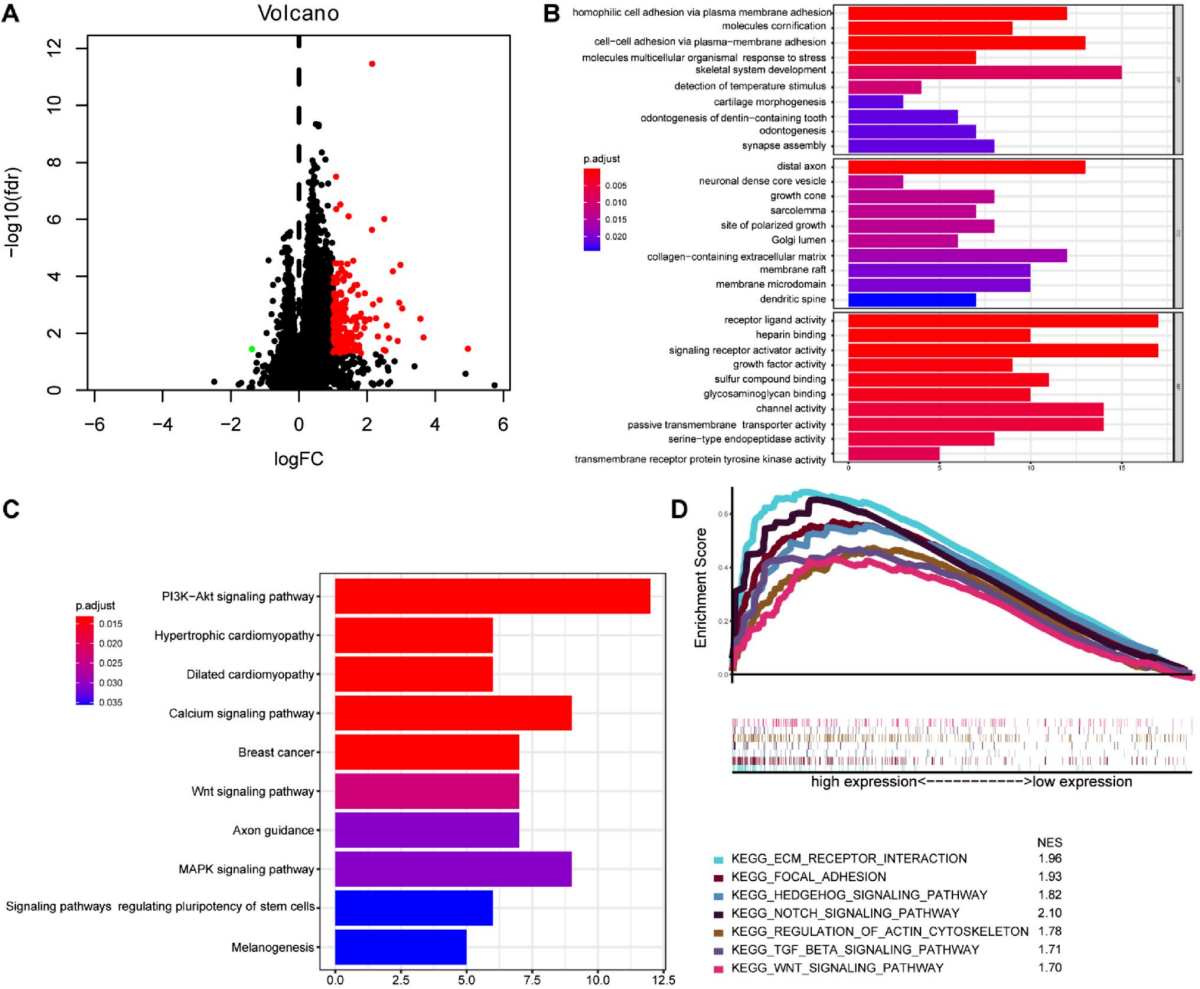
Analysis of pathway enrichment and functional annotation between high- and low-risk subsets. **A** Volcano plot of DEGs between the two risk subsets. **B** Gene ontology annotated for Treg-related classifier. **C** Enrichment analysis of KEGG pathway. **D** GSEA between high- and low-risk subpopulations

To investigate the biological function of the Treg-related signature, the analyses of GO, KEGG, and GSEA were conducted. The heatmap showed 185 differentially expressed genes (DEGs), selected by the R package “limma”, between risk groups (Fig. 10A). Enriched biological processes (BPs) were mainly concentrated in homophilic cell adhesion via plasma-membrane adhesion, molecules cornification, and cell–cell adhesion via plasma-membrane adhesion. In cellular components (CCs) analysis, these DEGs were enriched in the growth cone, site of polarized growth, and collagen-containing extracellular matrix. The molecular functions (MFs) indicated these DEGs were associated with receptor–ligand activity, signaling receptor activator activity, and growth factor activity (Fig. 10B). The KEGG pathways that were enriched in these DEGs were PI3K − Akt signaling pathway, calcium signaling pathway and Wnt signaling pathway (Fig. 10C). Then, a functional enrichment analysis of these DGEs was performed between risk groups. GSEA indicated that the pathways profoundly enriched in the high-risk group were ECM receptor interaction, focal adhesion, notch signaling pathway, hedgehog signaling pathway, regulation of actin cytoskeleton, TGF-beta signaling pathway, and WNT signaling pathway, while no significant pathways concentrated in the low-risk group (Fig. 10D). A complete list of enriched pathways can be found in Supplementary Table 3.

### Correlation between Treg-related classifier and tumor-immune microenvironment (TIME) and treatment responses

**Fig. 11:**
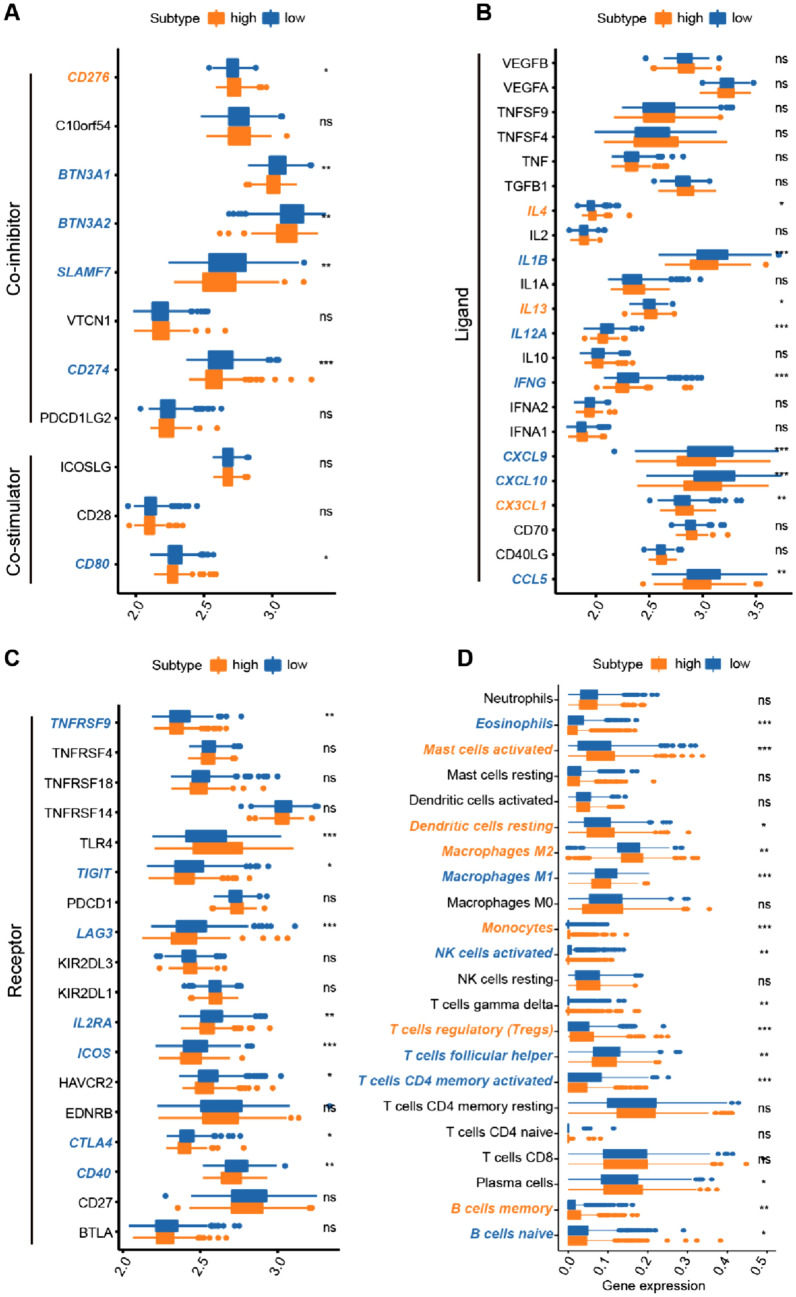
Correlation between Treg-related classifier and tumor-immune microenvironment (TIME). **A–C** The comparison of immunomodulators between risk groups was visualized in bar plot. **D** The bar plot revealed the 22 immune cell subpopulations between different risk groups

Due to the close relationship between Treg-related classifiers and immunomodulators (such as co-inhibitors, co-stimulators, ligands, and receptors), immune cells have a profound impact on the prediction of clinical outcomes and treatment effectiveness (Fridman et al. 2017; Thorsson et al. 2018). We further examined the difference and relationship between immunomodulators and these immune cells with risk groups. In terms of immunomodulators, the expression of co-stimulators (CD80), co-inhibitors (BTN3A1, BTN3A2, CD274, and SLAMF7), ligands (IL1B, IL12A, INFG, CXCL9, CXCL10, and CCL5), and receptors (TNFRSF9, TIGIT, LAG3, IL2RA, ICOS, CTLA4, and CD40) was elevated in the low-risk group, whereas the expression of CD276, IL4, IL13, and CXCL1 was significantly downregulated in the low-risk group, compared with those in the high-risk group (Fig. 11A–C). The CIBERSORT results revealed that the abundance of Tregs (T cells regulatory) was significantly higher in the high-risk group compared with those in the low-risk group. We also found that the fractions of other immune cells, including Mast cells activated, monocytes, dendritic cells resting, B cells memory, and macrophages M2 were significantly increased in high-risk patients than in low-risk patients, whereas the expression levels of B cells naïve, T cells CD4 memory activated, T cells follicular helper, T cells gamma delta, NK cells activated, macrophages M1, and eosinophils were significantly lower in the high-risk group (Fig. 11D). Furthermore, the risk score was positively associated with subpopulations of Mast cells activated, dendritic cells resting, B cells memory, monocytes, macrophages M2, and Tregs, while negatively related to subpopulations of B cells naive, eosinophils, T cells gamma delta, NK cells activated, T cells follicular helper, macrophages M1, and T cells CD4 memory activated (Supplementary Fig. 5).

**Fig. 12:**
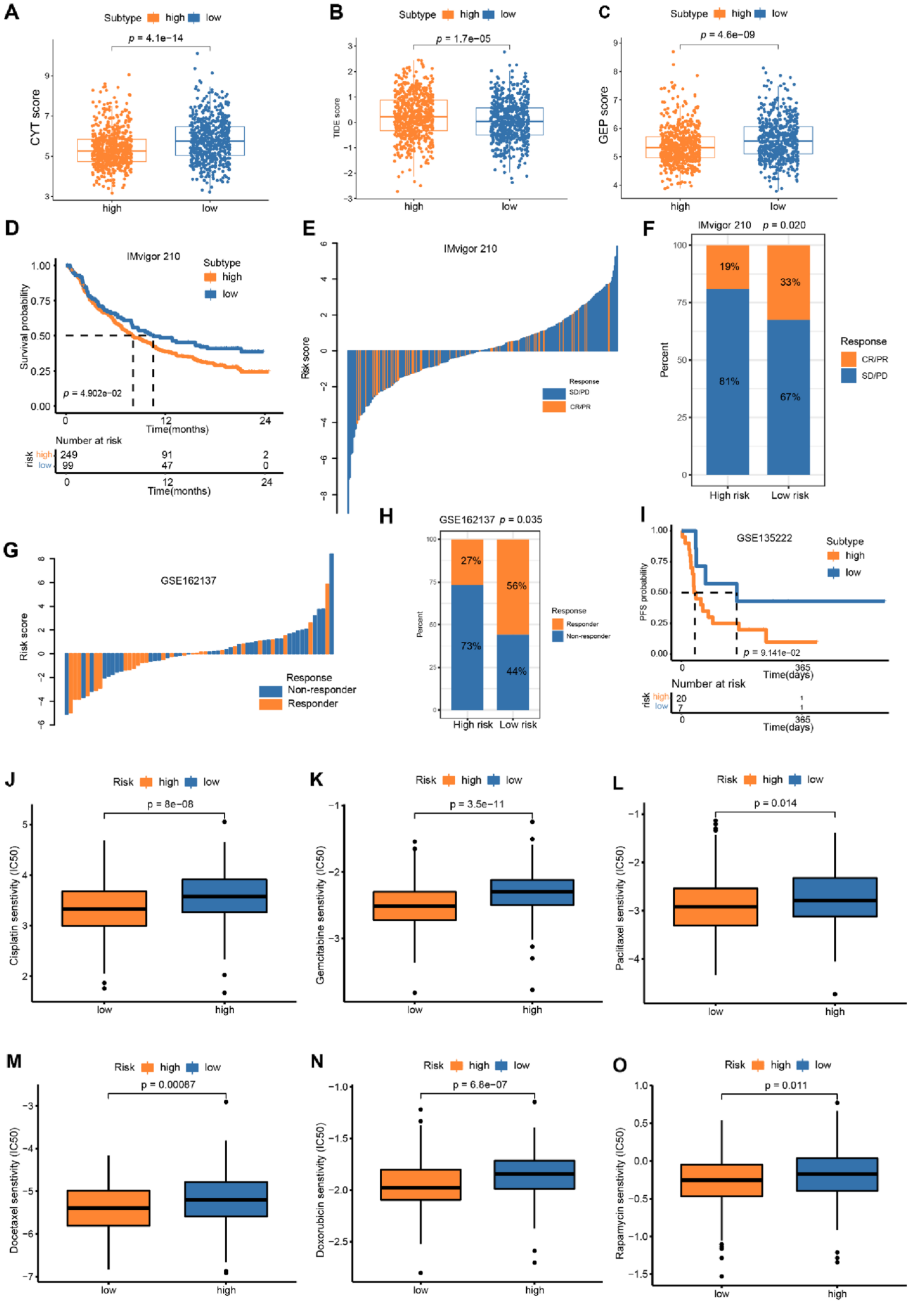
Correlation between Treg-related classifier and immunotherapy and chemo-agents therapeutic response. **A–C** The difference of CYT, GEP, and TIDE scores between risk groups. KM survival curves of different risk groups in IMvigor 210 cohort (**D)** and GSE13522 **(I)**. The Waterfall plot of Treg-score for different therapeutic response groups in IMvigor 210 set **(E)** and GSE16137 set **(G)**. Rate of therapeutic response to anti PD-1/PD-L1 immunotherapy in high- or low-Treg-score subsets in the (**F)** IMvigor210 cohort and **(H)** GSE13522 (Chi-square test, *P* < 0.05). The comparison of IC50 of six common chemotherapies (**J** cisplatin; **K** gemcitabine; **L** paclitaxel; **M** docetaxel; **N** doxorubicin; **O** rapamycin) between risk groups

Several analyses were performed to test the significance of our Treg-related classifier in predicting the efficacy of immunotherapy and chemotherapy. We first conducted the correlation between the risk groups and two promising biomarkers (GEP and CYT) to predict the effectiveness of immunotherapy. Our results revealed that patients within the low-risk group in the entire validation cohort had significantly higher CYT and GEP scores than those in the high-risk group (Fig. 12A, B). Moreover, the TIDE algorithm was also conducted to predict the response to immunotherapy. The results showed that patients in the high-risk group had elevated TIDE scores than those in the low-risk group, which might have lower immunotherapy efficacy (Fig. 12C).

Next, the association between Treg-related classifier and ICI (immune checkpoint inhibitor) therapeutic efficacy were performed in IMvigor210 set, GSE135222 set, and GSE162137 set. We applied our Treg-related classifier to count risk score of each patient among these sets, then divided them into high-risk group and low-risk group according to the optimal cutoff value calculated by ROC curve. In IMvigor210 set, we found that patients in the low-risk group were significantly correlated with a better OS for patients with PD-1/PD-L1 blockade therapy and a higher percentage of CR/PR than those in the high-risk group (Fig. 12D–F). Similarly, the response rate of low-risk group was higher than high-risk group in GSE135222 set (56.0% vs. 27%, *P* = 0.035; Fig. 12G, H). Moreover, survival analysis revealed that low-risk group patients had longer PFS (*P* = 0.091; Fig. 12I). In addition, we assessed the chemotherapy response of CC patients with different risk groups. Our results indicated that patients in the low-risk group had significantly lower IC50 values of six chemotherapy agents compared to those in the high-risk group (Fig. 12J–O). Overall, the Treg-related classifier exhibited a promising predictive ability for selecting patients which could benefit from ICI therapy and chemotherapy.

## Discussion

Colon cancer is one of the leading causes of cancer mortality worldwide. When patients receive an initial diagnosis, approximately 60% are in local advanced stages (stage II/III), and even after successful resection, there is a 20–30% risk of recurrence (Ju et al. 2019). Therefore, accurate risk stratification for stage I–III colon cancer patients is the key to the postoperative treatment strategy. The results showed that general-stage ladders could not fully predict prognosis for some colon cancers. The TNM staging cannot clearly distinguish the prognosis of patients with stage I–III colon cancer, especially in patients receiving adjuvant chemotherapy. Their 5-year overall survival is 50–90% (Brenner et al. 2014).

Targeting immune-infiltrating cells has recently gained significant attention as opposed to the direct killing of tumor cells. Intra-tumoral Treg is a heterogeneous cell population in colon cancer, which has a potential impact on the prognosis of patients (Zhang et al. 2015). There is some evidence that the immunosuppressive properties of Treg may facilitate the escape of tumor cells from anti-tumor immunity in the early stages of inflammation-related tumors. Treg can hinder the occurrence and development of tumors by inhibiting the inflammatory response (Erdman et al. 2005). However, the role of Tregs in the prognosis of colon cancer has been controversial. Nakagawa et al. (Nakagawa et al. 2015) revealed that Treg is associated with an optimal prognosis, while others have shown that Tregs in tumors indicate a poor prognosis (Saito et al. 2016; Sideras et al. 2018). As a result, a Treg-related risk model has been developed as a novel tool to predict recurrence-free survival after stage I–III colon cancer diagnosis.

We abstracted five cohorts from TCGA and GEO in this study that included a total of 1,194 patients with stage I–III CC. The patients were divided into a training cohort, two validation cohorts, and an entire validation cohort. First, 22 types of immune cell fractions were evaluated using the CIBERSORT web portal, and then we verified a significant grey module and 4813 candidate genes related to Treg using WGCNA analysis. A nine Treg-related gene signature was constructed using univariate Cox, Lasso, and multivariate Cox analyses. The model could divide CC patients into high- and low-risk groups with distinct recurrence-free survival in multiple cohorts (all *p* < 0.05). We also observed that the risk scores were significantly associated with several clinical factors, including recurrence status, N status, T stage, and TNM stage. As recurrence after postoperative, positive lymph node metastasis, higher T stage, and TNM stage were common prognostic indicator for poor clinical outcomes of colon cancer (Babcock et al. 2018; Huang et al. 2021a, b; Huang et al. 2021a, b; Bananzadeh et al. 2022), we can speculated that these factors would associated with higher risk scores, which is consistent with our results. To enhance its use in clinics, a nomogram including traditional clinical parameters and risk signatures was developed for CC. A ROC, C-index, and calibration curve demonstrated its robust predictive ability. Meanwhile, KM analysis revealed a significant difference in the subgroup analyses' survival, especially in different TNM stages and with adjuvant chemotherapy.

According to the National Comprehensive Cancer Network (NCCN) and Chinese Society of Clinical Oncology (CSCO) guidelines, adjuvant chemotherapy is a standard treatment for part of stage II and III patients (Diagnosis et al. 2019; Benson et al. 2021). However, some patients were still unable to benefit from adjuvant chemotherapy, resulting in rapid recurrence and metastasis. This Treg-related classifier demonstrates the ability to predict the recurrence-free survival (RFS) not only for patients with postoperative colon cancer patients, but also for patients with varying TNM stages I–III during subgroup analysis. Furthermore, this model possesses the capability to discern the specific demographic that is more inclined to derive advantages from adjuvant therapy, as anticipated. Consequently, our studies indicated that this prognostic nomogram predicts recurrence-free survival in stage I–III colon cancer, which could assist in identifying high-risk colon cancer patients who need more aggressive treatment.

Furthermore, GO enrichment analysis revealed that the prognostic Treg-related genes were mainly involved in critical cellular processes, such as cell adhesion, receptor–ligand activity, and growth factor. For the KEGG pathway analysis, the Wnt, Notch, Hedgehog (HH), and TGF-BETA signaling pathways were included in the KEGG-enriched pathways. The imbalance of the Wnt pathway is one of the important reasons for the occurrence and development of colon cancer (Malki et al. 2021). Its activated downstream proliferation signal is involved in the deterioration of colon cancer (Cheng et al. 2019). Several studies have demonstrated that the Wnt pathway regulates cancer adaptation and innate immunity in the tumor microenvironments. Infiltration and function of T cells were considered suppressed by Wnt signaling (Luke et al. 2019; Cane et al. 2021). Van et al. (2013) showed that the immunosuppressive function of Treg cells was limited by Wnt-β-catenin signaling inhibiting Foxp3 transcriptional activity via TCF-1-dependent manner. The NOTCH pathway is highly conserved and is widely involved in the occurrence and development of malignant tumors, including colon cancer (Vinson et al. 2016). The Notch pathway is involved in tumor development and regulates T cell development, maintenance, and activation (Tsukumo et al. 2004; Samon et al. 2008; Auderset et al. 2013). Optimal T-cell-mediated anti-tumor immunity requires NOTCH signaling (Tchekneva et al. 2019). TMEs and tumor cells resist T-cell-mediated killing by inhibiting the Notch signaling pathway. The effect of the Notch pathway on TME is reflected mainly in the reduction of the sub-population of myeloid-derived suppressor cells (MDSCs), TAMs and Tregs after inhibiting the activity of the Notch pathway (Mao et al. 2018). Bertrand et al. (2012)summarized that crosstalk exists between WNT, Notch, Hedgehog and TGF-BETA in colon cancer. TGF-β signaling promotes EMT through WNT, HH, and Notch. The Hedgehog signaling plays a vital role in intestinal carcinogenesis and its TME. A strong body of research indicates that cancer-associated fibroblasts (CAFs) and inflammatory factors in the TME, such as interleukin 6 (IL-6) and interferon-g (IFN-g), macrophages, and T cell-dependent immune responses, affected tumor growth through the HH signaling pathway (Zhang et al. 2021). These results suggest that a Treg-related classifier is associated with cancer occurrence, progression, and immune response and may be a potential biomarker to predict clinical outcomes.

The tumor-immune microenvironment is crucial in cancer biology (Hanahan et al. 2011). Several studies report that tumor-infiltrating immune cells (TILs) are important in the development, progression, and chemotherapeutic efficacy of colon cancer. Evidence shows that high Treg infiltration is associated with a poor prognosis in multiple cancers, including colon cancer (Takeuchi et al. 2016; Soo et al. 2018). Given our classifier based on Treg-related genes, the Treg-related classifier produced a risk score consistent with expectations and positively related to the quantity of Tregs. Moreover, patients within the high-risk group were associated with poor RFS, and the infiltration levels of Tregs were significantly higher in the high-risk group. Furthermore, we found that some cancer-promoting TILs, such as M2 macrophages, monocytes, and mast cells, increased in the high-risk group. For example, substantial evidence suggests that colorectal cancer patients with higher M2-Macrophages have poorer clinical outcomes (Yin et al. 2017; Wei et al. 2019; Xue et al. 2021). Lan et al. reported that exosomes secreted by M2 macrophages contributed to colorectal cancer cells’ migration and invasion in vivo and in vitro (Lan et al. 2019). Furthermore, low-risk patients possessed a higher fraction of some tumor-suppressor TILs (such as NK cells activated, Macrophages M1 (Chanmee et al. 2014), and T cells CD4 memory activated (Tay et al. 2021)), associated with improved RFS. NK cells exert their cytotoxic function against cancer cells and their abundance is correlated with improved clinical outcomes (Eckl et al. 2012). M1 macrophages performed their anti-tumor ability via releasing cytokines and chemokines (Mantovani et al. 2015; Mantovani et al. 2017; Parisi et al. 2018), and the higher abundance of M1 macrophages predicted favorable survival (Ma et al. 2010). These data suggest that the Treg-related classifier risk score was positively linked with subpopulations of inhibitory immunity cells (such as Treg and M2 macrophage), whereas it was negatively correlated with immune-activated subpopulations (M1 macrophages and NK cells activated), indicating the immunosuppressive condition of the high-risk subset with matching inferior RFS. Moreover, three well-known algorithms (CYT, GEP and TIDE) were employed to assess the predictive ability of Treg-related classifier in immunotherapy. Our findings suggest that patients in the low-risk group trend to benefit from immunotherapy. These findings were well verified in the three immunotherapy cohorts. Namely, low-risk patients had an obviously better clinical outcome than high-risk patients, indicating that they may benefit from immune checkpoint immunotherapy. Moreover, we observed that low-risk patients were more likely to sensitive to these six common chem-agents.

We have constructed a Treg-related risk model with good predictive ability for RFS for colon cancer stage I–III. There are also some limitations to this study. First, to incorporate more data into our research, we have selected as many data sets as possible in the GEO database. Fusing multiple data may increase the possibility of over-correction in data processing. In addition, we only repeatedly verified the effectiveness of the model through several queues, but further experimental validation is required to determine whether these genes represented in the model are involved in the progression of stage I–III CC and how they alter the phenotypes of Treg cells. These results of this study are valuable and promising for future research.

## Conclusions

In summary, based on the multiple data sets, we constructed a risk prediction model correlated to Tregs in patients with stage I–III CC through various bioinformatic methods. Furthermore, our study was more effective and accurate as numerous training and validation queues were designed, particularly in different databases. A nomogram was developed using the signature and traditional clinical parameters, to predict clinical outcomes and assist in clinical procedures. Our findings indicate that this classifier may indicate immunotherapy and chemotherapy response, allowing for a more targeted selection of patients who may benefit from these treatments.

### Supplementary Information

Below is the link to the electronic supplementary material.

Supplementary file1 (DOCX 18 KB)

Supplementary file2 (DOCX 19 KB)

Supplementary file3 (XLSX 10 KB)

Supplementary file4 (TIF 683 KB)

Supplementary file5 (TIF 2029 KB)

Supplementary file6 (TIF 3914 KB)

Supplementary file7 (TIF 1700 KB)

Supplementary file8 (TIF 1060 KB)

## Data Availability

The data of TCGA-COAD cohort were downloaded from the UCSC Xena database (http://xena.ucsc.edu/). The data of GES39582, GSE37892, GSE33113, GSE17536, GSE135222, and GSE162137 were downloaded from the Gene Expression Omnibus (GEO) database (http://www.ncbi.nlm.nih.gov/geo/). The data of IMvigor210 were obtained from online (http://research-pub.gene.com/IMvigor210CoreBiologies).
