# Supplementary material for: A prognostic nomogram for predicting recurrence-free survival of stage I–III colon cancer based on immune-infiltrating Treg-related genes
Source: J Cancer Res Clin Oncol. 2023 Jul 27;149(15):13523–43. doi: 10.1007/s00432-023-05187-y (PMC10590341; doi:10.1007/s00432-023-05187-y)
Supplement: Supplementary file 1 — Supplementary file1 (DOCX 18 KB) [file 432_2023_5187_MOESM1_ESM.docx]

**Supplementary Table1 The total of published signatures was retrieved from the literatures.**

| **Model** | **PMID** | **Author** | **SYMBOL NAME** | **Coef** |
| --- | --- | --- | --- | --- |
| Model1 | 32423860 | Dai Q | RARRES3 | NA |
| Model1 | 32423860 | Dai Q | PLA2G2A | NA |
| Model1 | 32423860 | Dai Q | EPB41L3 | NA |
| Model1 | 32423860 | Dai Q | IFITM2 | NA |
| Model2 | 29377588 | Dai W | ACTR3B | - 0.052 |
| Model2 | 29377588 | Dai W | BLMH | - 0.116 |
| Model2 | 29377588 | Dai W | CCL20 | - 0.047 |
| Model2 | 29377588 | Dai W | CMPK2 | - 0.121 |
| Model2 | 29377588 | Dai W | ECM1 | 0.259 |
| Model2 | 29377588 | Dai W | GZMB | 0.043 |
| Model2 | 29377588 | Dai W | HES6 | - 0.287 |
| Model2 | 29377588 | Dai W | IL7 | - 0.102 |
| Model2 | 29377588 | Dai W | KLK10 | 0.201 |
| Model2 | 29377588 | Dai W | KRT6A | - 0.015 |
| Model2 | 29377588 | Dai W | MMP9 | - 0.302 |
| Model2 | 29377588 | Dai W | MSLN | 0.038 |
| Model2 | 29377588 | Dai W | OAS1 | - 0.217 |
| Model2 | 29377588 | Dai W | PUS7 | - 0.236 |
| Model2 | 29377588 | Dai W | ZNF426 | - 0.168 |
| Model3 | 25749516 | Teodoro V | ABCA1 | NA |
| Model3 | 25749516 | Teodoro V | ACSL1 | NA |
| Model3 | 25749516 | Teodoro V | AGPAT1 | NA |
| Model3 | 25749516 | Teodoro V | SCD | NA |
| Model4 | 30933267 | Mo S | CAPN2 | 0.795 |
| Model4 | 30933267 | Mo S | ATG16L2 | -0.079 |
| Model4 | 30933267 | Mo S | TP63 | 0.407 |
| Model4 | 30933267 | Mo S | SIRT1 | -0.324 |
| Model4 | 30933267 | Mo S | RPS6KB1 | -0.412 |
| Model4 | 30933267 | Mo S | PEX3 | -0.009 |
| Model4 | 30933267 | Mo S | ATG5 | 0.126 |
| Model4 | 30933267 | Mo S | UVRAG | -0.018 |
| Model4 | 30933267 | Mo S | NAF1 | -0.096 |
