# Supplementary material for: A prognostic nomogram for predicting recurrence-free survival of stage I–III colon cancer based on immune-infiltrating Treg-related genes
Source: J Cancer Res Clin Oncol. 2023 Jul 27;149(15):13523–43. doi: 10.1007/s00432-023-05187-y (PMC10590341; doi:10.1007/s00432-023-05187-y)
Supplement: Supplementary file 2 — Supplementary file2 (DOCX 19 KB) [file 432_2023_5187_MOESM2_ESM.docx]

**Supplementary Table2 Univariate Cox regression analysis in different sets**

| **Signatures** | **Univariate analysis** | |
| --- | --- | --- |
|  | **HR (95% CI)** | ***P* value** |
| **Training set (n =485)** |  |  |
| Nomogram | 3.049 (2.221-4.188) | ＜0.001 |
| Risk score | 2.610 (1.991-3.421) | ＜0.001 |
| Dai Q | 2.718 (1.093-6.759) | 0.031 |
| Dai W | 2.926 (2.315-3.699) | ＜0.001 |
| Teodoro V | 2.718 (1.554-4.756) | ＜0.001 |
| Mo S | 2.083 (1.425-3.044) | ＜0.001 |
| **The 1^st^ test set (n =392)** |  |  |
| Nomogram | 1.101 (1.067-1.135) | ＜0.001 |
| Risk score | 1.459 (1.135-1.876) | 0.003 |
| Dai Q | 2.718 (1.511-4.886) | ＜0.001 |
| Dai W | 1.810 (1.412-2.321) | ＜0.001 |
| Teodoro V | 2.718 (1.755-4.210) | ＜0.001 |
| Mo S | 1.464 (0.977- 2.192) | 0.065 |
| **The 2^nd^ test set (n = 317)** |  |  |
| Nomogram | 1.308 (1.116-1.534) | ＜0.001 |
| Risk score | 1.601 (1.241- 2.065) | ＜0.001 |
| Dai Q | 2.715 (0.732- 10.067) | 0.135 |
| Dai W | 1.001 (0.995- 1.006) | 0.847 |
| Teodoro V | 3.045 (1.583-5.854) | 0.014 |
| Mo S | 0.998 (0.989- 1.008) | 0.697 |
| **The total test set (n =1194)** |  |  |
| Nomogram | 1.466 (1.358-1.583) | ＜0.001 |
| Risk score | 2.181(1.707-2.787) | ＜0.001 |
| Dai Q | 2.718 (1.171-6.312) | 0.020 |
| Dai W | 2.267 (1.906-2.696) | ＜0.001 |
| Teodoro V | 2.718 (1.834-4.027) | ＜0.001 |
| Mo S | 1.725 (1.294-2.299) | ＜0.001 |
