# Supplementary figures and images for: A prognostic nomogram for predicting recurrence-free survival of stage I–III colon cancer based on immune-infiltrating Treg-related genes
Source: J Cancer Res Clin Oncol. 2023 Jul 27;149(15):13523–43. doi: 10.1007/s00432-023-05187-y (PMC10590341; doi:10.1007/s00432-023-05187-y)

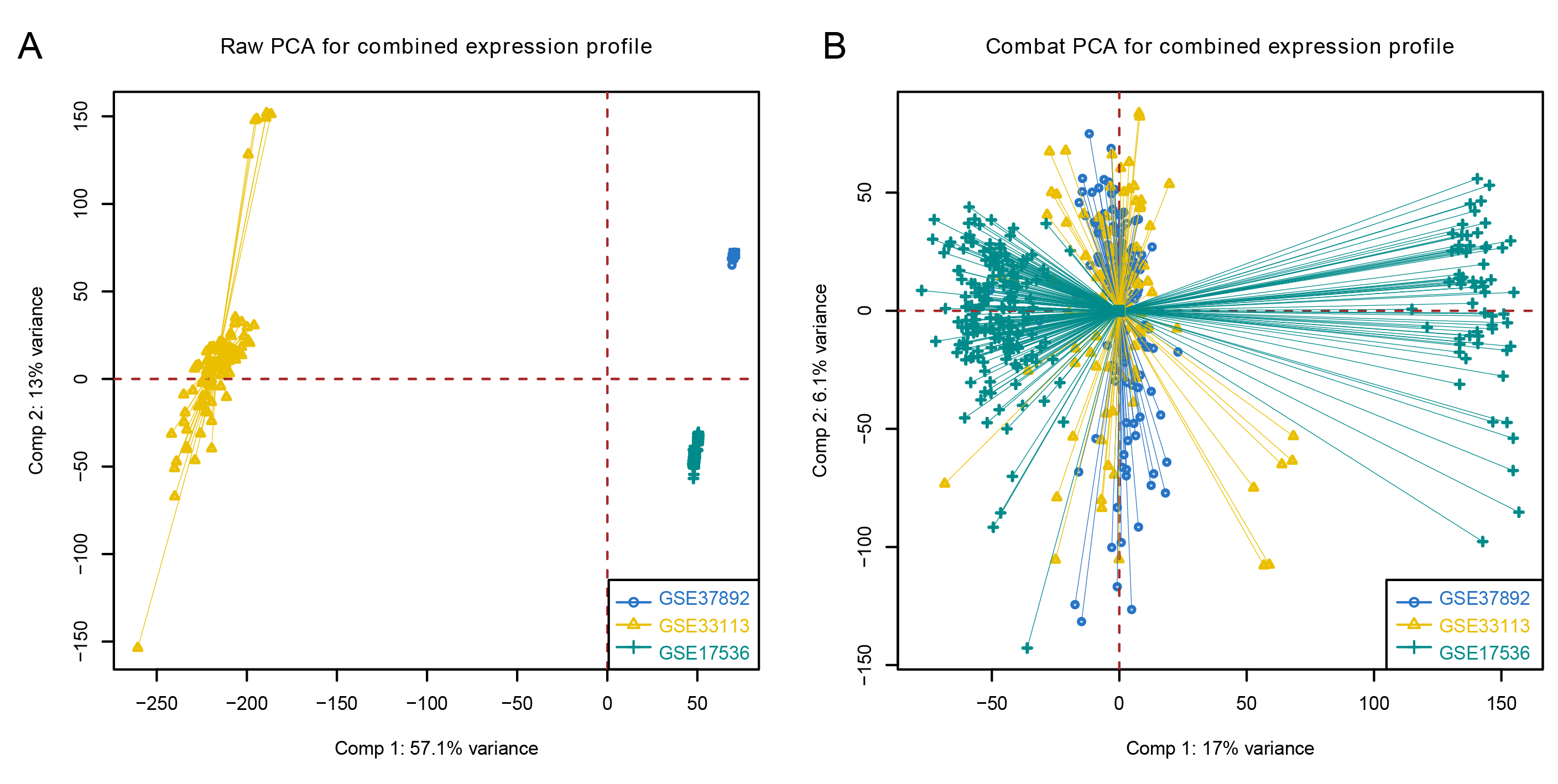

Supplement: Supplementary file 4 — Supplementary file4 (TIF 683 KB) [file 432_2023_5187_MOESM4_ESM.tif]

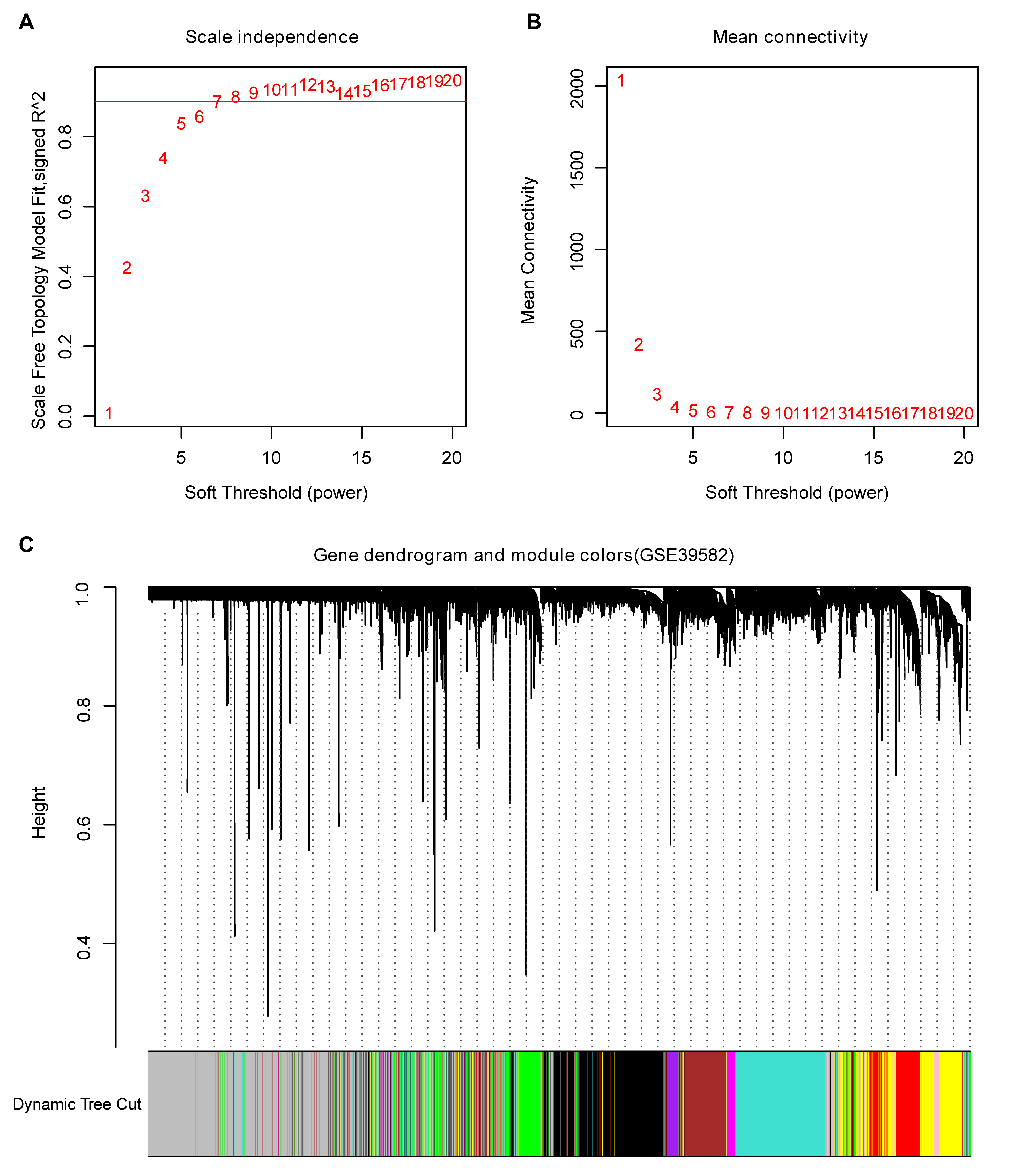

Supplement: Supplementary file 5 — Supplementary file5 (TIF 2029 KB) [file 432_2023_5187_MOESM5_ESM.tif]

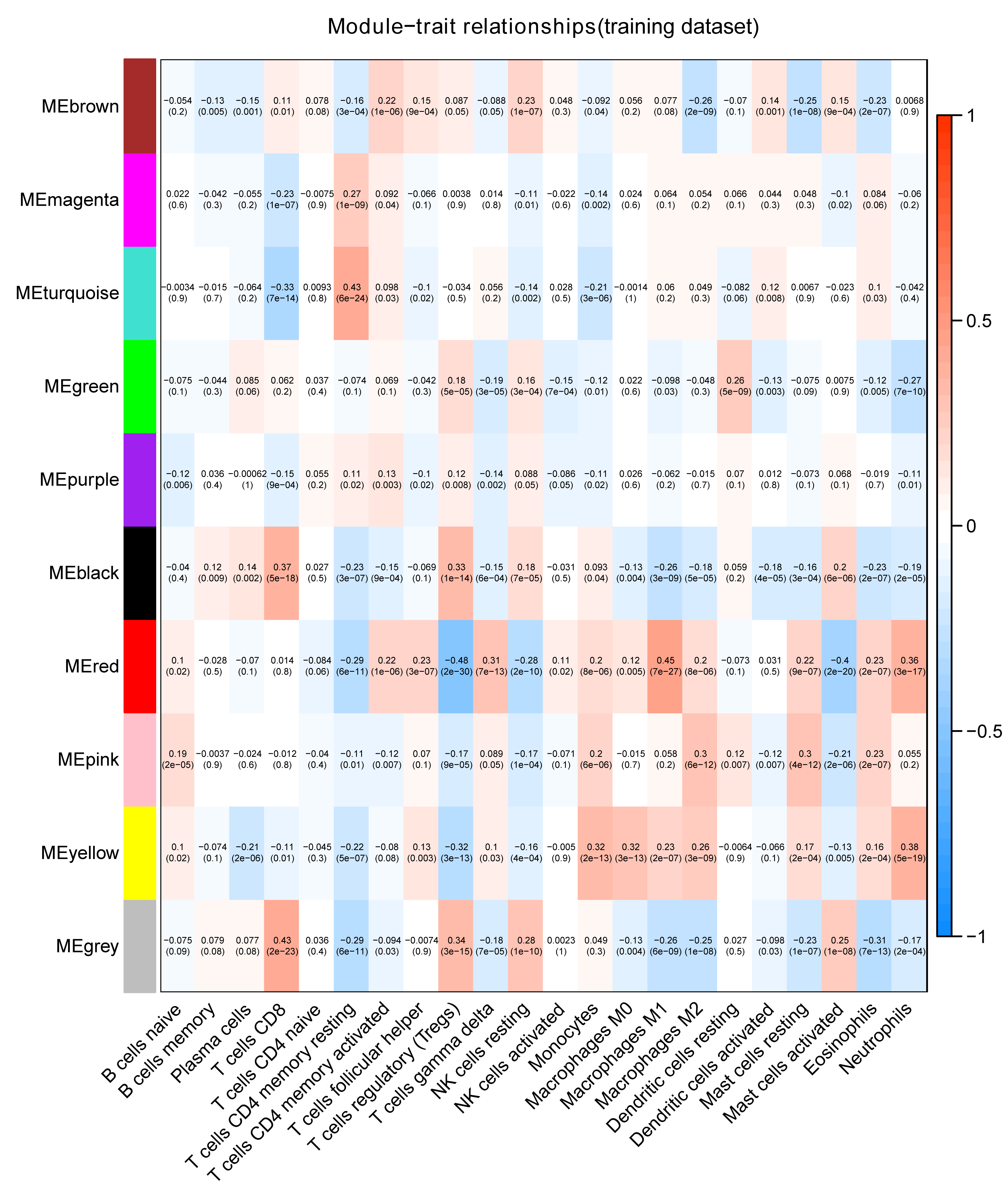

Supplement: Supplementary file 6 — Supplementary file6 (TIF 3914 KB) [file 432_2023_5187_MOESM6_ESM.tif]

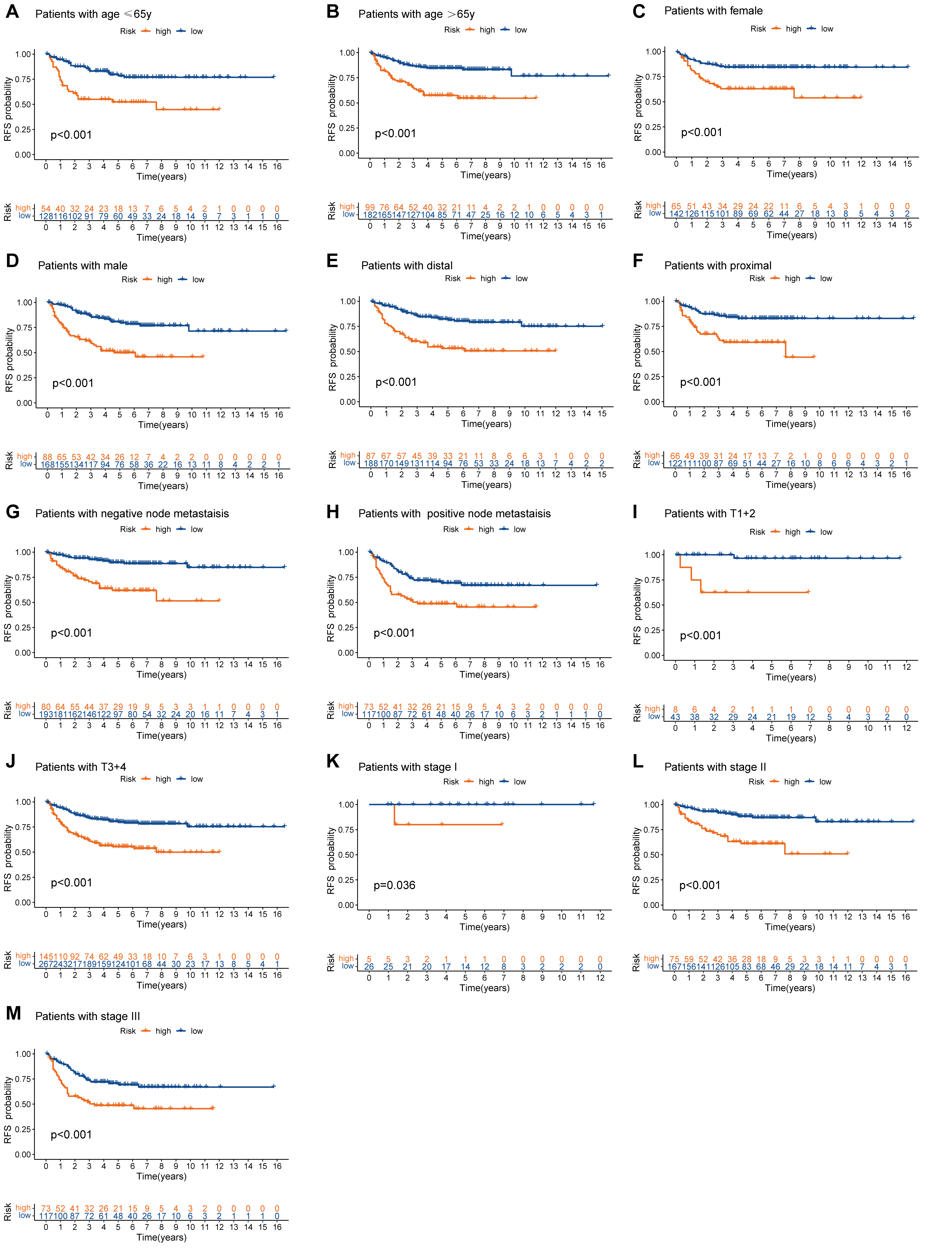

Supplement: Supplementary file 7 — Supplementary file7 (TIF 1700 KB) [file 432_2023_5187_MOESM7_ESM.tif]

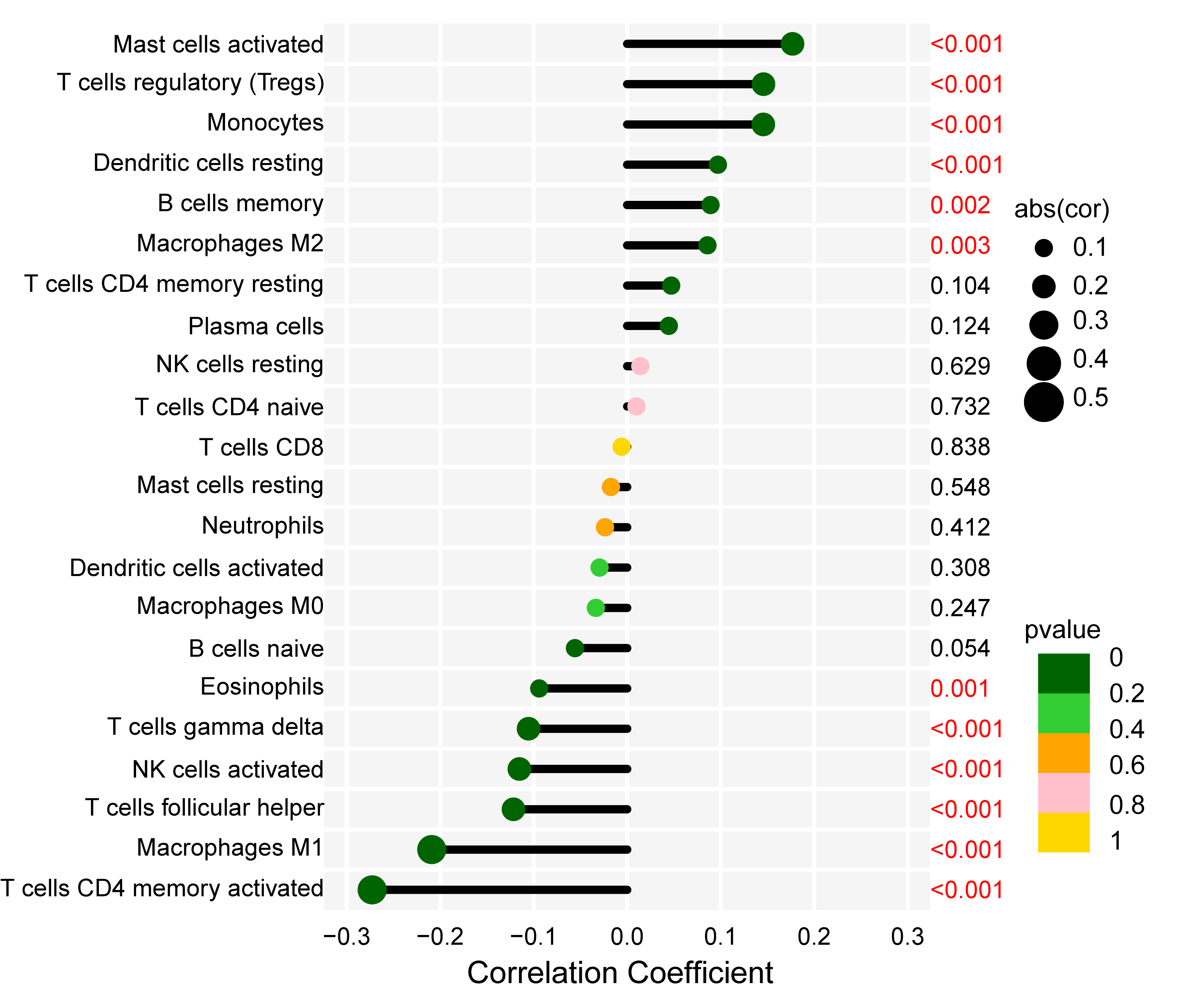

Supplement: Supplementary file 8 — Supplementary file8 (TIF 1060 KB) [file 432_2023_5187_MOESM8_ESM.tif]
